# A Review of Functional Encryption in IoT Applications

**DOI:** 10.3390/s22197567

**Published:** 2022-10-06

**Authors:** Khurram Shahzad, Tanveer Zia, Emad-ul-Haq Qazi

**Affiliations:** 1 School of Computing, Mathematics and Engineering, Charles Sturt University, Wagga Wagga 2650, Australia; 2 Center of Excellence in Cybercrime and Digital Forensics, Naif Arab University for Security Sciences, Riyadh 14812, Saudi Arabia

**Keywords:** IoT, functional encryption, security, privacy, fog and cloud computing, data sharing, blockchain, e-Health

## Abstract

The Internet of Things (IoT) represents a growing aspect of how entities, including humans and organizations, are likely to connect with others in their public and private interactions. The exponential rise in the number of IoT devices, resulting from ever-growing IoT applications, also gives rise to new opportunities for exploiting potential security vulnerabilities. In contrast to conventional cryptosystems, frameworks that incorporate fine-grained access control offer better opportunities for protecting valuable assets, especially when the connectivity level is dense. Functional encryption is an exciting new paradigm of public-key encryption that supports fine-grained access control, generalizing a range of existing fine-grained access control mechanisms. This survey reviews the recent applications of functional encryption and the major cryptographic primitives that it covers, identifying areas where the adoption of these primitives has had the greatest impact. We first provide an overview of different application areas where these access control schemes have been applied. Then, an in-depth survey of how the schemes are used in a multitude of applications related to IoT is given, rendering a potential vision of security and integrity that this growing field promises. Towards the end, we identify some research trends and state the open challenges that current developments face for a secure IoT realization.

## 1. Introduction

### 1.1. Background

**Figure 1:**
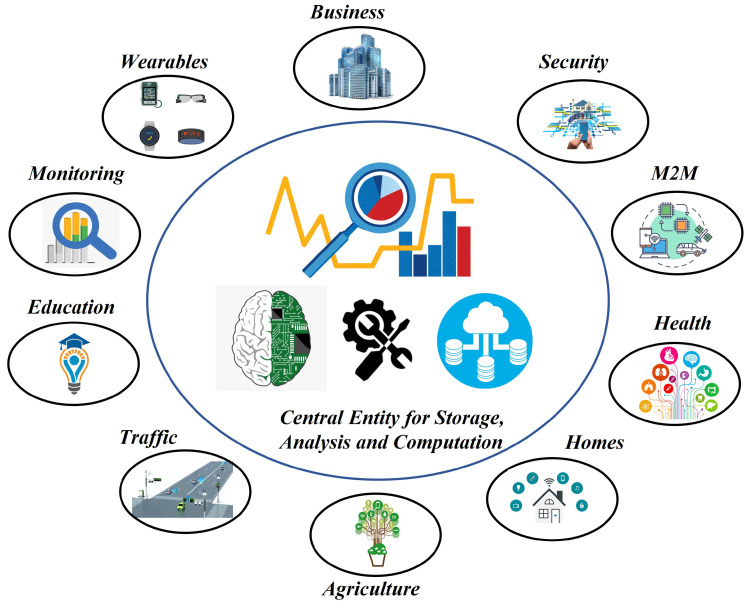
Overview of IoT applications.

The last decade has seen an explosion in digital technologies, essentially transforming the way we produce, store and transmit information. Our lives are dependent on the use of services brought about through this change, whether it be as simple a task as opening the garage door or transferring highly personal information including medical records and financial transactions. One of the drivers behind the recent data revolution is the Internet of Things (IoT) [1], which revolves around the idea of connecting a multitude of devices. These devices, ranging from small sensors to advanced electronic gadgets, communicate and exchange information with either each other or central entities to form a network. Through the use of smart terminals equipped with sensors and actuators, and the integration of technologies and promising solutions, the IoT constitutes a network of distributed intelligence, realizing the vision of a smart connected world. The variety of applications wherein IoT has made a difference is vast, ranging from massive IoT applications to time- and information-critical IoT networks. The range of IoT applications is visually depicted in Figure 1. The positives of this modern digital transformation also resulted in some negatives, among which the security and privacy of information appears to be of the highest concern. Apart from the standard security concerns involved in generic broadcast-natured wireless systems, IoT comes with its own additional challenges. These challenges stem from its unique characteristics including the range of communication, desired capabilities of self-organization and the availability of limited resources. The IoT also often connects to the cloud for added storage and computation capabilities, which brings upon additional challenges in maintaining desired security and privacy.

Researchers from the industry and academia alike are interested in analyzing and developing ways to achieve the highest possible degree of information security, whether it be identity protection, data integrity and security, or simply ensuring users that both their day-to-day and sensitive information is in safe hands. Traditional approaches to data integrity and security are highly dependent on public-key cryptography [2,3], which has been driving the research efforts in the cryptographic community for a long time. In these conventional cryptosystems, the decryption process eventually recovers the underlying plaintext if the decryption process is successful, which means that *all* the information contained in the plaintext is revealed. However, there are many applications where only a partial exposure of the contained information is needed. For example, a financial organization may want to filter transactions above a certain amount in which case decrypting the exact value of all transaction records is not needed. Rather, a simple decision on the transaction amount determining whether it is above or below the desired threshold is needed. Similarly, access rights in broadcast transmissions need to determine the level of access that a user is granted instead of revealing the complete content of the transmission. These rights can be determined based on, among other things, a user’s identity, affiliation, attributes and organizational standing. This is specifically needed to hold under circumstances where the encryptor does not necessarily know the identity or attributes of the decryptor, but rather all that is needed are the required attributes to determine the access structure. Such a structure for data access drastically changes the way that information access can be determined since it fundamentally alters the way in which data are distributed among receivers and also affects the steps taken to ensure the security and integrity of these data. This fine-grained access control provides a method of controlling certain forms of data access, and compared to generalized data access control, it uses more distinctive and variable methods for allowing access. Fine-gained access control provides the ability to centrally store data, maintain confidentiality and precision, improve security and improve the information access for authorized users.

Functional encryption (FE) [4] is a public-key encryption scheme with different decryption keys allowing a user to learn specific functions of the encrypted data. The control that FE offers over which functions are allowed to be computed on the data and by which user immensely benefits the data owner in multiple ways. For example, if suspicious activity is observed within an organization, a scan of system logs might help understand the origin of this activity. However, sharing the complete logs with an external security expert may not be feasible as it gives them access to the entire network data. In such a situation, a function can be generated for the expert to only look at the transmission-control protocol (TCP) port, giving the external expert the corresponding key, and restricting their access only to the desired information. Since its introduction, functional encryption has attracted a lot of interest, and its known results are broadly categorized as either focusing on feasibility results for general functionalities, or concrete, efficient realizations for restricted functionalities of practical interest [5]. In this work, we review the recent applications of functional encryption and the major cryptographic primitives that it covers. We identify the areas in which the adoption of these primitives has had the greatest impact, especially in the realm of IoT. Our work provides a review of these applications without strictly going into the mathematical details associated with each area of application. This essentially helps readers understand the domains and guides them towards further exploration in the desired directions.

### 1.2. Related Works

A number of studies have recently been presented in the literature which have surveyed the different aspects of fine-grained access control schemes, including attribute and identity-based encryption, although the applications of FE have not been considered on the larger scale. Lee et al. [6] presented a study on attribute-based encryption (ABE) schemes for access control in cloud environments. The study is broken down based on two different access policy structures and two encryption schemes, namely ABE, key-policy ABE (KP-ABE), ciphertext-policy ABE (CP-ABE), hierarchical ABE (HABE), and ABE with non-monotonic access structure. The authors provide a detailed performance and security analysis of the considered schemes and conclude that user accountability is hard to justify in these schemes. Furthermore, due to a pre-defined access structure in these schemes, all the encrypted data need to be regenerated if a new user wants to access data and their attributes are not in the access structure.

Based on the use of mobile devices to access private data hosted in the cloud and the physical limitations of the mobile device to perform complex computations, Moffat et al. [7] analyzed the CP-ABE approaches to data security of the mobile devices in a recent survey. The authors found that the computational demands of CP-ABE encryption and decryption is inefficient on mobile devices due to their physical limitations including the processor and battery power as well as network bandwidth. To overcome these shortcomings, some solutions have been proposed, including managing the efficiency and complexity of encryption-generated computations, delegating encryption to assisting nodes, and efforts to enhance the physical traits of these devices. The authors in [8] surveyed the various varieties of ABE for use in cloud environments, making observations about their use and provision of access privilege. A comparison of different ABE techniques based on their distinctive features such as computation overhead, user revocation, resistance to collusion, and attribute association was also provided.

Zhao et al. [9] provided a comprehensive survey of the applications of identity-based cryptography (IBC) in mobile ad hoc networks (MANETs). The authors observed that although IBC offers properties suitable for use in MANETs including the generation and storage of privacy keys and the elimination of any need to distribute and store partner certificates, IBC requires system parameters to be distributed among all communicating parties before any encryption/decryption could take place, putting the “ad hoc” property out of scope. The authors in [10] provided a review of IBC and a comparison with traditional public-key encryption. After reviewing some important IBE schemes based on bilinear pairing, a computational primitive widely used to build various identity-based cryptographic schemes, a number of real-world applications were identified. Li et al. [11] surveyed identity-based signcryption (IBSC) schemes (Signcryption is the cryptographic primitive that meets both requirements of authenticity and confidentiality of crowdsourced data among users and is ideal for ensuring secure authentic data storage and transmission in industrial crowdsourcing environments), providing a comparison on their security properties and efficiency. Several recommendations to improve the performance of IBSC were made, including the construction of schemes in the standard model, the construction of post-quantum signcryption schemes and efficiency improvements.

Wang et al. [12] provided a survey on the two main techniques of searchable encryption (SE), namely symmetric SE (SSE) and public key encryption with keyword search (PEKS). Different SE schemes are categorized and compared in terms of functionality, efficiency, and security. The work of [13] describes the notion of SE in the context of healthcare applications and characterizes the SE use cases into different healthcare scenarios. The authors provided a comprehensive overview of the four representative SE techniques: SSE, PEKS, attribute-based encryption with keyword search (ABKS), and proxy re-encryption with keyword search (PRES) according to different electronic health records (EHRs) retrieving scenarios and requirements. Furthermore, the categorization and comparison of different SE schemes in terms of their security, efficiency, and functionality is also provided. In a more recent work, the authors in [14] presented a complete taxonomy/classification of the searchable encryption schemes in terms of the type of search, type of index, results retrieved, implementation type, multiplicity of users, and the techniques used. A more recent study in [15] provided the reader with a wide view of the different FE schemes, focusing on their functionalities, limitations, security models, and the involved mathematical assumptions. The authors also presented an overview of “*non-standard*” FE schemes that go beyond the inner product encryption and schemes with enhanced properties.

### 1.3. Methodology, Contribution and Organization

#### 1.3.1. Research Methodology

As discussed in Section 1.2, the abundant literature on different aspects of fine-grained access control in general, and related to IoT in particular, has been previously analyzed from various perspectives. This work aimed to complement the previous works by providing a comprehensive review of the different access control approaches combined under the umbrella of functional encryption, as specifically applied to IoT applications. To achieve the desired objectives, we adopted the Preferred Reporting Items for Systematic Reviews and Meta-Analyses (PRISMA) methodology, a framework developed to support systematic reviews and meta-analyses of literature [16]. To approach the problem at hand, we performed a search, in November 2021, in the Scopus database using the available search tool. Furthermore, we performed another search in April 2022 using the same database to include any new works published in early 2022. We chose this database owing to its breadth and relevancy in regard to the literature, as it includes the majority of the related archives and journals. Furthermore, this database is frequently used in a lot of such academic reviews.

**Figure 2:**
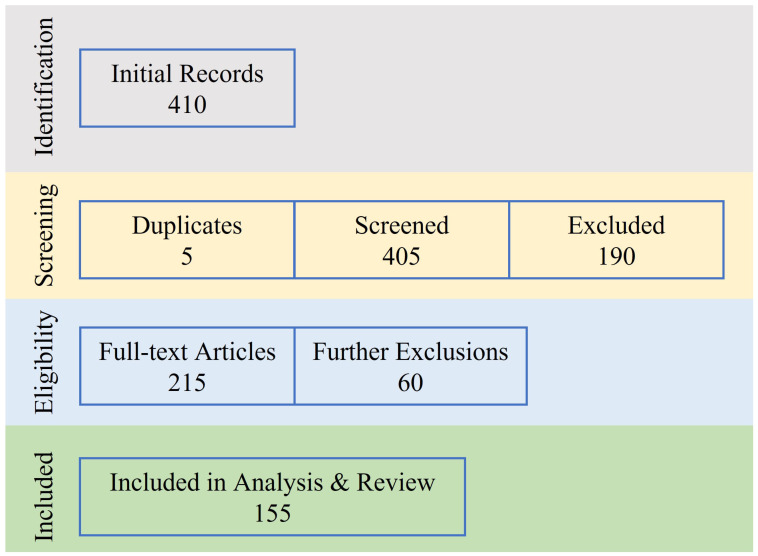
PRISMA components of this review on applications of fine-grained access control in IoT.

**Figure 3:**
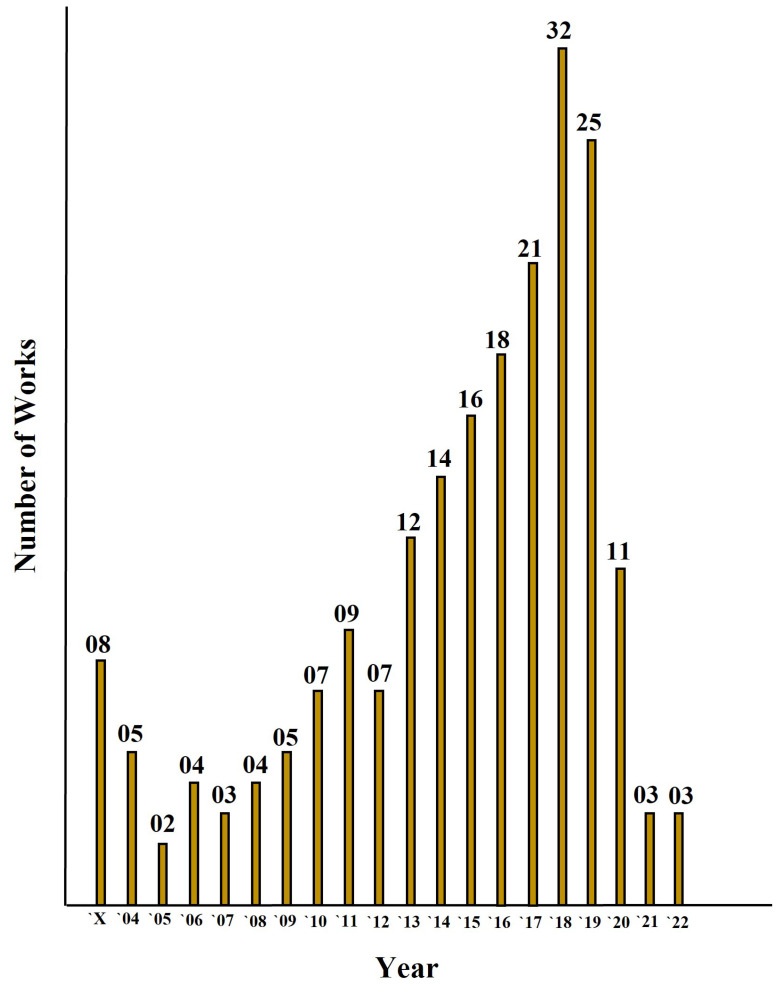
The number of works considered in this work, shown according to the publication year. ‘X represents year 2003 and earlier.

The approach which we considered for search through the database looked for terms that comprised of different word combinations describing the problem at hand. The terms “Functional Encryption”; “Attribute based Encryption”, “Identity based Encryption”, “Searchable Encryption” and “Predicate Encryption” were used in combination with terms relating to “Internet of Things” and underlying applications. Once the articles that resulted from the search were extracted, a manual review of all the results was performed through focusing on the title, abstract, keywords and main text of the papers, eliminating any works that were deemed unrelated to the purpose at hand. Any duplicate results were also eliminated at this stage. It should be noted here that any articles that discussed the aforementioned variations of fine-grained access control in mathematical detail but did not address specific IoT applications were also excluded from further analysis. More specifically, the articles considered for further analysis met the criteria as (i) they considered fine-grained access control in one of the variations given above; and (ii) they presented application of access control in an application area of IoT. Further screening was also applied to those records that did not offer full-text availability and were deemed unsuitable for inclusion in the review due to the fact they were not applicable in IoT areas. We summarize the process of search, exclusion, and selection in Figure 2, where it can be seen that although the initial search resulted in a total of 410 articles, the number was reduced to 155 articles after the analysis, filtering, and classification. We also provided a distribution of the works considered in this study according to the publication year in Figure 3.

#### 1.3.2. Contribution

In this work, we surveyed the recent applications of FE along with the commonly occurring cryptographic primitives that it generalizes. More specifically, we looked at the prominent works presented in the literature in recent years covering the applications of these cryptographic primitives to IoT-related domains. Our main contributions can be summarized as follows:

- We present a comprehensive review of the use of FE and encompassed fine-grain access control mechanisms in IoT applications.
- We provide a detailed overview of the different application areas where fine-grain access schemes were applied.
- We provide an in-depth survey of how these schemes are used in a multitude of applications related to IoT. The aim was to provide the reader with a potential vision of fine-grained security and integrity in IoT.
- We identify some research trends and state some open challenges that current developments face for a secure IoT realization.

#### 1.3.3. Paper Organization

**Figure 4:**
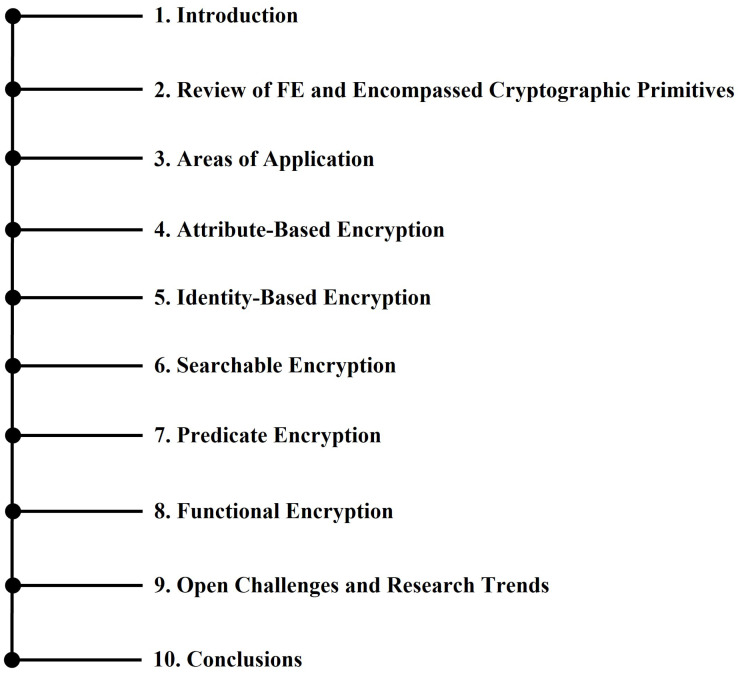
Outline of this work.

The basics of considered cryptographic primitives and early development works are described in Section 2.1, Section 2.2, Section 2.3, Section 2.4 and Section 2.5, whereas Section 3 gives a brief description of the considered application areas. The different applications that we cover in this work range from IoT and cloud applications to data sharing and classification and machine learning. The presented work is divided into sections pertaining to different techniques, wherein Section 4 considers the applications of ABE; Section 5 describes the work utilizing IBE for the most common application areas; Section 6 presents the works using SE; Section 7 describes the applications of PE; and the applications wherein FE is utilized are given in Section 8. We provide some open challenges and related research trends in Section 9, and finally, some conclusions are drawn in Section 10. A graphical outline of this work is also shown in Figure 4. We also note that there is a significant overlap in the application areas of all these cryptographic techniques, whereas the underlying schemes and targeted benefit drawn upon differ greatly depending on the utilization of these schemes.

## 2. Review of Functional Encryption and Encompassed Cryptographic Primitives

FE is a strong generalization of several existing cryptographic primitives which addresses fine-grained access control in varying aspects. In this section, we first present these underlying primitives and then provide some details on FE before delving into the application areas of interest. Towards the end of this section, we also described how the following presented cryptography schemes can be constructed from FE.

### 2.1. Attribute-Based Encryption

Attribute-based encryption (ABE), first proposed by Sahai and Waters [17], is a type of public-key cryptosystem where the secret key of a user and the associated ciphertext are linked to their attributes. As a result, the decryption of a ciphertext is only possible if the set of attributes of the user’s key match the attributes of the ciphertext. The following set of cryptographic primitives are commonly used in ABE schemes.

- (MK,EK) = *Setup*(λ)—this primitive initializes the cryptographic scheme, where taking λ as the input security parameter, a master key MK and an encryption key EK are generated.
- CP = *Encrypt*(*M*, P, EK)—This primitive encrypts the message/plaintext *M* under the policy P using the encryption key EK, generating the ciphertext CP.
- DK = *KeyGen*(γ, MK)—This primitive generates the decryption key DK taking into account the user attributes contained in γ and the master key MK.
- *M* = *Decrypt*(CP,DK)—This primitive outputs the message *M* taking as input the ciphertext CP and decryption key DK if the decryption is successful. In the case of failure, it outputs ⊥ (the symbol ⊥ is commonly used in the literature to denote a failure to decrypt).

Owing to the fact that attributes are crucial entities in any ABE scheme, two classifications of ABE named CP-ABE and KP-ABE were proposed, which have been widely adopted in the literature.

#### 2.1.1. Cihpertext-Policy ABE

CP-ABE, as first proposed by Bethencourt et al. [18], offers sufficient flexibility since it allows rules that specify *which* private keys can decrypt *which* ciphertexts. These private keys are associated with sets of attributes or labels, and when a user encrypts, they encrypt to an access policy which specifies which keys will be able to decrypt. CP-ABE has been deemed more appropriate in the literature for data sharing systems since data owners make and own the decision concerning the access policy. For CP-ABE, since the policy is embedded in the ciphertext itself, the access structure for CP-ABE answers the question: “*Who can access the data that I am encrypting?*”.

A variety of CP-ABE schemes have been proposed for flexible access control policies. Cheung et al. [19] developed the first CP-ABE scheme under the decisional bilinear Diffie–Hellman (DBDH) assumption and used positive and negative attributes under AND-gate constructions. One drawback of this work was that the size of ciphertext and secret key linearly increased with the number of attributes. Goyal et al. [20] and Liang et al. [21] made further improvements to CP-ABE through a flexible access structure which supported AND, OR and *threshold* operations. Using non-interactive cryptographic assumption, Waters [22] proposed a new CP-ABE scheme where access the structure is presented using a linear secret sharing scheme, however, it suffered the same problem of linear increase in encryption and decryption overhead with the access structure.

#### 2.1.2. Key-Policy ABE

KP-ABE was originally proposed by Goyal et al. [23] which, based on the decisional bilinear Diffie–Hellman (DBDH) assumption, allowed fine-grained access to monotone structures. In the proposed scheme, the secret key is associated with a pre-accessed structure where the user is only able to decrypt the ciphertext if the attribute set satisfies the access structure in the secret key. For KP-ABE, since the policy is embedded inside the key belonging to the decryptor, the access structure of KP-ABE simply answers the following question: “*As a decryptor, what type of data can I access?*”.

### 2.2. Identity-Based Encryption

Public-key cryptography enables the communicating parties to encrypt/decrypt messages and send them through insecure network channels. However, before secure communication can be enabled, users must generate encryption and signature key pairs, be verified by a certificate authority (CA) and receive CA-signed certificates. Furthermore, key management issues including the key storage capacity required to archive all the private keys for distinct users and key certification and validation processes [24,25] result in major drawbacks of the public-key cryptography’s practical implementation. The idea of identity-based cryptography was first proposed by Shamir in 1984 [26], putting forth the notion of using a unique string such as a user’s name, email address or contact number to explicitly compute the user’s private key. This enabled a new paradigm providing a key-certificate-less platform effectively overcoming the issues plaguing public-key cryptography. However, it came to reality only after Boneh and Franklin designed the first secure and practical IBE scheme [27] using bilinear pairing on elliptic curves. In the same year, Cocks proposed an IBE scheme using quadratic residuosity as the underlying primitive [28]. Since then, other primitives including trapdoor subgroups [29,30] have also been proposed. A conventional IBE scheme can be described in the following cryptographic primitives:

- (MK,Par)=Setup(λ)—Upon the input of security parameter λ, it outputs public system parameters Par, and master key MK. The Par are released to the public while MK is kept secret.
- PK=Extract(MK,Par,ID)—Upon the input of MK, Par and user’s identity string ID, this generates the user’s corresponding private key PK.
- CP=Encrypt(Par,ID,M)—Upon the input of Par, ID and message *M*, this outputs the ciphertext CP.
- M=Decrypt(Par,ID,CP)—This outputs the message *M* taking as input the ciphertext CP alongwith Par and ID. In the case of decryption failure, it outputs ⊥.

### 2.3. Searchable Encryption

The diverse set of ciphertexts that are stored in the cloud servers or exchanged for information and analysis among IoT-based sensor entities need to be searched and located efficiently. The inability to access encrypted files limits the flexibility and precision of data retrieval, which might result in insufficient or incorrect search results. A simple solution may be to store the encrypted data on the cloud and a follow a complete decryption protocol every time there is a search query that needs to be completed. However, this may result in increased difficulty and complexity levels for data processing and application. Some cryptographic primitives such as secure multi-party computation (SMC) [31] and fully homomorphic encryption (FHE) [32] were proposed for the restricted handling of encrypted data, however, these primitives are currently inefficient and hard to use in real environments. The work in [33] proposed a first SE scheme based on ciphertext scanning, which enables users to store encrypted data in the cloud, perform keyword searches through the ciphertext domain and selectively retrieve relevant documents from the cloud. The basic scheme of [33] is described as follows: Assume that Alice wants to encrypt a document that contains a sequence of words $W_{1},\cdots ,W_{l}$. Alice generates a sequence of pseudorandom values $S_{1},\cdots ,S_{l}$ using a pseudorandom generator *G*, where each $S_{i}$ is n−m bits long. To encrypt an n−bit word $W_{i}$, Alice takes the pseudorandom bits $S_{i}$, sets $T_{i}⊕\langle S_{i},F_{k_{i}}\left(S_{i}\right)\rangle$ and outputs the ciphertext $C_{i}⨁W_{i}⨁T_{i}$, where ⨁ denotes a direct sum. Different SE schemes have been proposed in the literature, including the works of Curtmola et al. [34] for optimal search time, Ibrahim et al. [35] with information retrieval system, Chen et al. [36] for security outsourcing of large-scale equation and Sun et al. [37] for the secure sorting of encrypted data.

### 2.4. Predicate Encryption

PE [38] is deemed a novel cryptographic primitive that provides accurate fine-grained access to encrypted data. In contrast to traditional public-key cryptographic systems, it has been widely adopted in point-to-point communication systems. A cryptographic system such as PE discerns the justification of information being only available to the users with access rights, providing more fine-grained control over the ciphertext. Predicate encryption has an associated attribute space A, a predicate space P and consists of the following algorithms:

- Setup(λ)—It takes as input the security parameter λ and outputs a public key PK, and master key MK.
- KeyGen(MK,f)—It takes as input the MK, and a predicate f∈P and outputs secret key $SK_{f}$.
- Encrypt(PK,I,M)—It takes as input a public key PK, an attribute I∈A and a message *M*, and outputs a ciphertext CP.
- $Decrypt(CP,SK_{f})$—It takes as input the ciphertext CP and a secret key $SK_{f}$, and outputs either a message *M*, or ⊥ in case of failure.

Two different types of PE have been proposed in the literature: asymmetric PE (ASPE) [38,39,40] and symmetric PE (SPE) [41,42,43], where the main difference in the two types lies in the identity of the searcher. In general, SPE is suitable for systems where the searcher is the same entity who has encrypted the data such as cloud storage systems, while in ASPE, the searcher is not necessarily the same entity as the data encryptor, such as email servers or credit card payment systems.

### 2.5. Functional Encryption

Traditional public-key encryption is deemed insufficient in a lot of emerging applications. For example, a decryption policy pertaining to the ciphertext under consideration needs to be specified, dictating that only the individuals who satisfy the policy can decrypt to obtain the plaintext. It is often necessary to only grant access to a function of the plaintext, depending on the decryptor’s authorization. For example, law enforcement agencies may ask the cloud to search for particular individual’s images, hence the cloud only needs restricted access to decrypt images that only contain the target individual, and nothing else from the images is revealed. Many applications such as spam filters, parental control, or targeted advertising, only require a partial knowledge of the data. Functional encryption adapts these useful applications for the desired data privacy and confidentiality, since only the relevant, processed information is revealed.

As compared to public-key cryptography systems, which contain three algorithms of Setup, Encryption and Decryption, FE systems also include a fourth algorithm called KeyGen. The KeyGen algorithm takes as input the master key mk generated by Setup and a description of some function *f*, and outputs a key sk[f] that is specific to the function *f*. More precisely, if *c* is the result of encrypting *x* with public key pk, then the decryption of *c* using sk[f] outputs f(x). It should be emphasized here that that sk(f) does not fully decrypt *c*, rather it provides only a function *f* of the full decryption. However, for full decryption, users can use a secret key for some function, say *g*, where g(x)=x for all *x*. An FE system is secure if an attacker with a set of secret keys $sk\left[f_{1}\right],\ldots ,sk\left[f_{t}\right]$ can learn nothing about the decryption of some ciphertext *c* other than what is revealed by the keys at the attacker’s disposal (we refer the interested reader to [4,15] for further mathematical details including the security model analysis).

**Figure 5:**
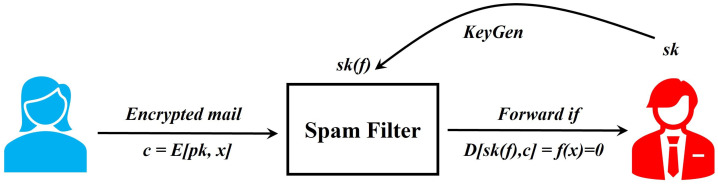
Functional encryption—spam filtering on encrypted mail.

For further illustration, we consider the common example of spam filtering on encrypted mail, as described in [44] and depicted in Figure 5. Here, the email recipient, who has a master secret key sk, gives a spam-filtering service a key sk[f] for the functionality *f*; this f satisfies f(x)=1 whenever message *x* is marked as spam by a specific spam predicate, otherwise f(x)=0. A sender encrypts an email message *x* to the recipient, but the spam filter blocks the message if it is spam. The spam filter does its job through the key sk[f] but learns nothing else about the contents of the message.

Based on the seminal work of [17], the tutorial style article by Boneh et al. [44] explains the basic ideas for FE, encompassing previous specializations of public-key encryptions such as IBE and ABE as special cases of FE. Motivating the use of FE through practical examples such as spam filtering on encrypted email, expressive access control and the mining of large datasets, it discusses how FE supports the richest possible families of functions and also provides an insight into the inherent limitations of FE systems. It is notable that FE generalizes several existing cryptographic primitives including IBE and ABE, among others. Under the FE terminology, IBE can be formulated as equality testing functionality, where assuming pk and mk to be output of an FE setup, the encryptor uses the encryption algorithm as E(pk,(id,m)) to obtain the ciphertext, while the data being encrypted are the pair (id,m). A recipient with an identity of $id^{*}$ can use the secret key $sk[f_{id^{*}}]$, issued by the authority, where the function $f_{id^{*}}$ outputs *m* if $id=id^{*}$, otherwise it produces a ⊥. Under this construction, users can only decrypt messages intended for $id^{*}$, otherwise they learn nothing about messages which are encrypted for other identities. Similarly, in the case of a ciphertext-policy ABE system, the policy ϕ specifies the recipient attributes that can decrypt the ciphertext and the encryption function takes into account the pair (ϕ,m) to generate the ciphertext. The message *m* is successfully decrypted if all the specified attributes of the recipient match the ones specified in the policy else a ⊥ is the output.

**Figure 6:**
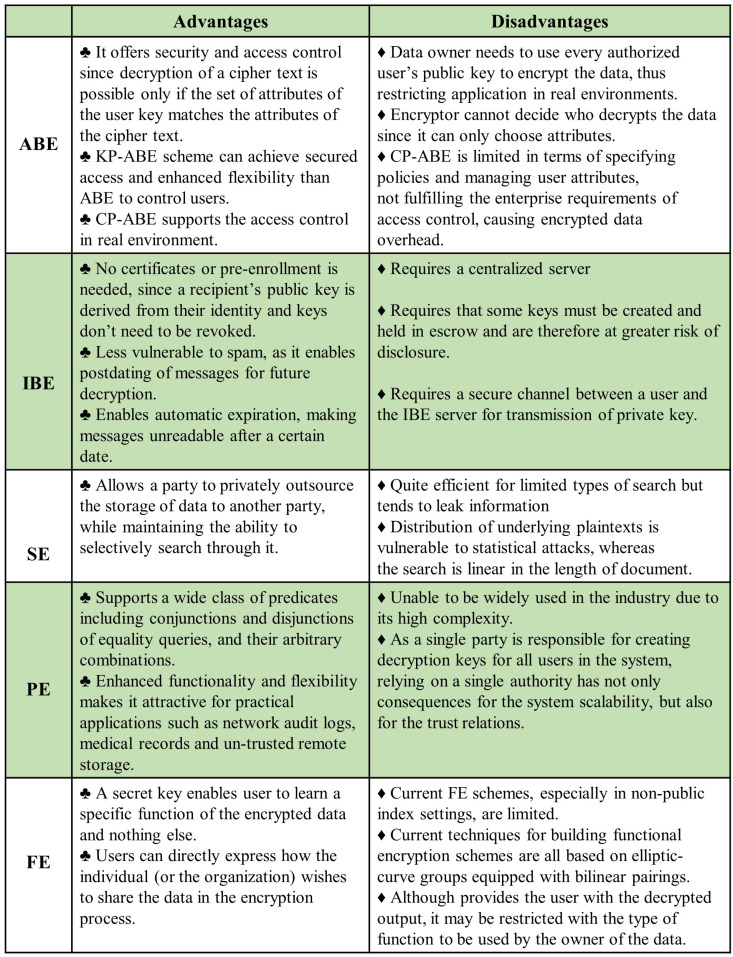
A brief summary of the advantages and disadvantages of cryptographic primitives considered in this work.

A brief summary of the advantages and disadvantages of the above discussed primitives is given in Figure 6, providing the reader with an intuitive understanding of these approaches. It should be noted that the details in Figure 6 are not application-specific, and can be used as general guidelines when choosing between different approaches to achieve fine-grained access control and security.

## 3. Areas of Application

In the modern day and age, the range of applications where the security and integrity of information plays a vital role is virtually unlimited. The amount of data exchanged in these applications is vast, and data integrity is not only important to the users but also to various service providers. The Internet of Things [1,45], enabling the realistic vision of *‘everything smart’* is one of the most common and crucial areas where the security and integrity of data are highly desirable. A variety of modern-day applications rely on numerous IoT devices distributed across every possible surface, equipped with various IoT-based sensors for data collection and further uploading these data to the relevant authorities. Apart from analysis and response, these data also allow control authorities to make intelligent decisions and develop algorithms for improved performance. Even though this realization of IoT seems exciting and able to solve several problems, the characteristics of these IoT sensor devices make the security and privacy of all the involved information very critical.

**Figure 7:**
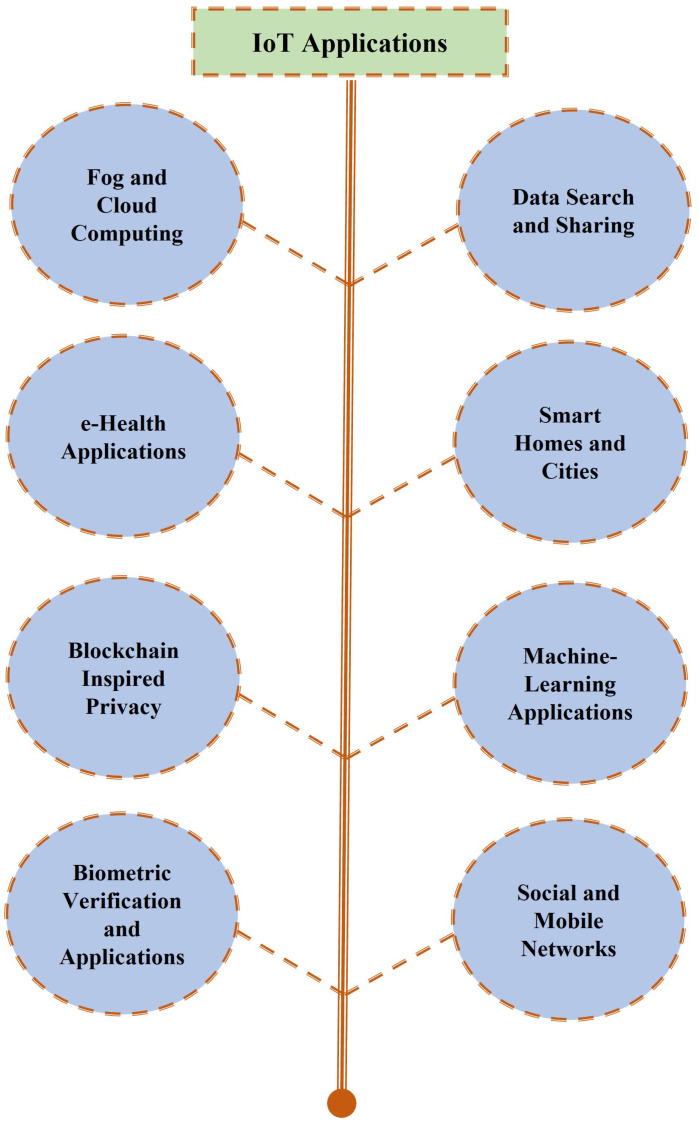
Application areas for FE and encompassed primitives considered in this work.

**Figure 8:**
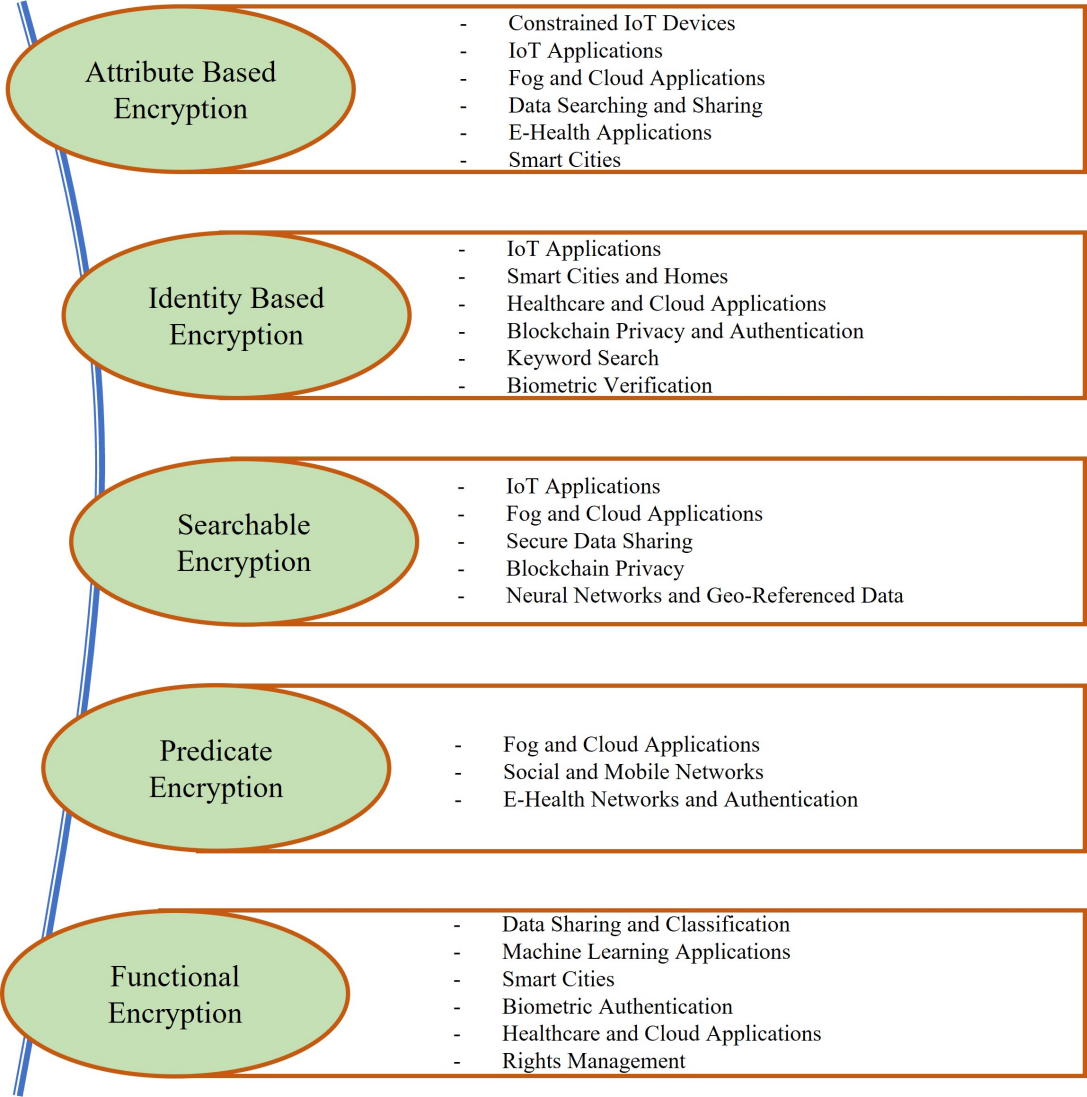
A list of applications covered in this work under considered cryptographic primitives.

In the following, we provide an overview of some of those application areas that were considered in this survey, providing the reader with a glimpse of relevant security concerns in these applications. The major application areas covered in this work are shown in Figure 7, whereas Figure 8 details the application areas considered under each cryptographic primitive. 

**Figure 9:**
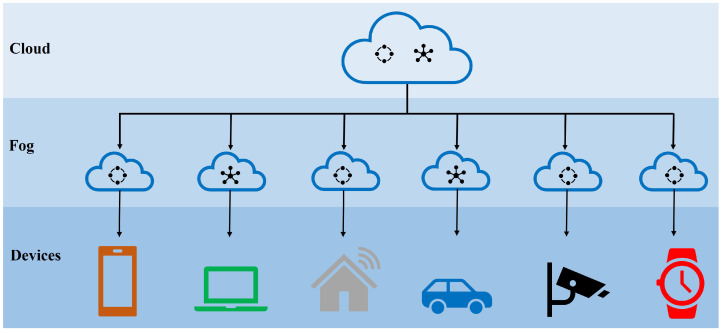
IoT data processing in cloud and fog.

**Fog and Cloud Computing**—Cloud computing [46] enables the sharing of resources as services for software, infrastructure and platforms for customers. Cloud customers, in general, store their sensitive data in encrypted form. Fog computing [47], as shown in Figure 9, extends cloud computing to the edge of the network, providing newer services such as location awareness, low latency and quality-of-service (QoS) enhancement. However, significant threats exist in cloud- and fog-based computing networks relating to data alteration, unauthorized access and eavesdropping attacks. Furthermore, fog nodes are considered to be more easily compromised and less trustworthy due to their closer deployment to the network edge. These issues raise the most concerns among users who look to utilize fog and cloud resources for data storage and sharing.

**Data Search and Sharing**—With the recent increase in the amount of data exchanged [48], their transmission and sharing across public networks should be treated carefully. This, in part, can be attributed to the fact that in public networks, participating users are not fully trusted and everyone, including the adversaries, can easily become part of the network. On the other hand, searching is an increasingly important aspect for the retrieval of desired information. Since data are generally stored in encrypted form with cloud service providers, extracting desired information may require the decryption of all the data which are computationally complex and infeasible. Furthermore, traditional public-key encryption with keyword search (PEKS) schemes involve public-key infrastructure (PKI) to authenticate users over the network which is considered a complex and costly task due to the involvement of certificate revocation, storage, distribution and verification. 

**Figure 10:**
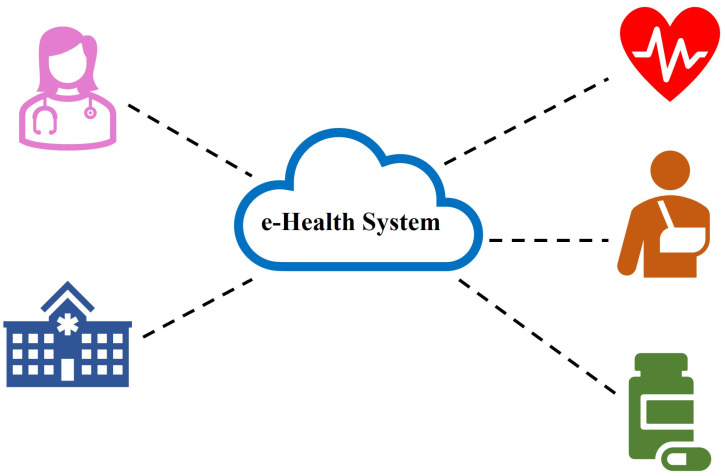
e-Health applications offer quick and safe access for both doctors and patients, resulting in better advice and services.

**e-Health Applications**—Smart health systems [49], as shown in Figure 10, enable the exchange of sensitive and personal data between doctors and patients. If the doctors have timely access to a patient’s medical information, this can result in better advice and medical services. The privacy of these data can be threatened if these sensitive data are exposed to an open network. Furthermore, since most health sensors and mobile devices have constrained resources, quickly producing and processing ciphertexts can be challenging. 

**Figure 11:**
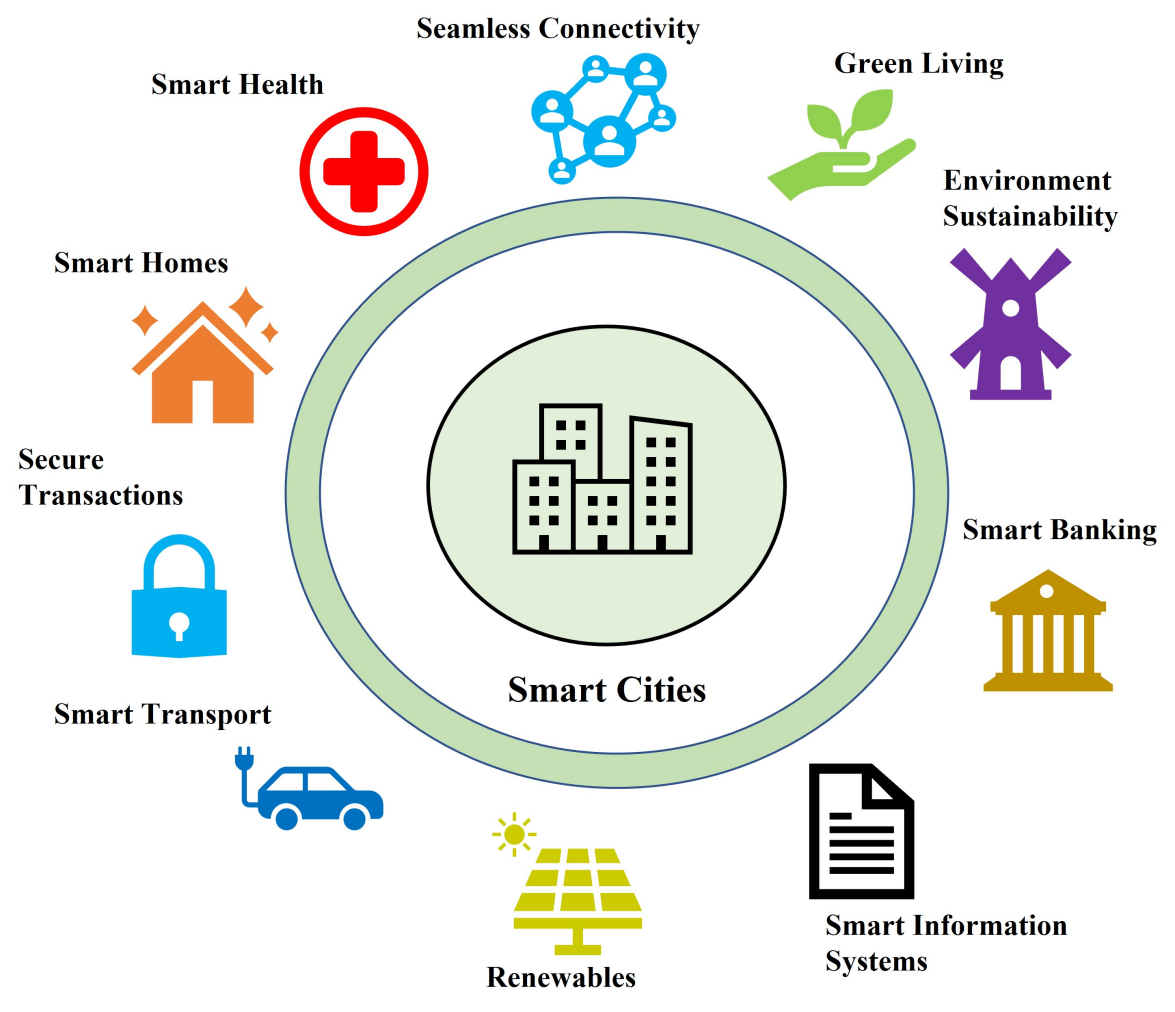
Smart cities are perceived to improve the overall quality of life for their citizens.

**Smart Homes and Cities**—Smart homes and cities, as shown in Figure 11, are perceived to facilitate the needs of modern citizens, improving the overall quality of their life. Smart homes [50] are envisioned to connect all appliances and objects together, integrating them in the Internet through smartphones and other mobile devices. However, all these connected devices contain personal information flowing through them, causing concerns among home owners about their privacy. On the other hand, smart cities [51] can address the major problems in most urban areas including traffic congestion, energy and resource management, education, sanitation and healthcare services. However, these technologies heavily rely on some underlying infrastructure, e.g., IoT sensors to achieve the desired purpose. Furthermore, the large amounts of data produced for the purposes of observation and analysis are generally outsourced to a cloud storage service (CSS) for ease of access and enhanced security. As soon as the data land on the cloud, the user’s control is lost and they have to fully trust the CSS. To encounter these problems, it has been often proposed to store encrypted data on the cloud while ensuring fine-grained access control.

**Blockchain-Inspired Privacy**—Blockchain is a comprehensive technology encompassing data storage, cryptography and distributed systems, among others, providing schemes to develop decentralized trust relationships [52,53]. This allows users to communicate and trade directly in the network instead of relying on third-party intermediaries. The distributed structure ensures that a compromise of one node in the chain does not affect the rest of the system. With the development of blockchain technology, its applications have transitioned from crypto-currencies to real economies. As security vulnerabilities make IoT devices an easy target for distributed denial-of-service attacks, malicious attackers and data breaches, blockchain technology can improve the security and scalability of IoT networks due to its transparency, truthfulness, immutability, and privacy features. On the other hand, in permissioned blockchain networks, each node can be owned by different organizations, without having to build a centralized network and to bring a certain level of trust among untrusting parties. 

**ML Applications**—Modern-day applications heavily rely on machine learning (ML) techniques [54]. In the realm of network privacy and security, these can be used to help detect and prevent malicious activity and finding any security vulnerabilities. In the case of an attack on ML engines, training sets or datasets, the result may range from breaking the ML model to adversely affecting the prediction and classification results. Furthermore, neural networks which play the role of building blocks in ML applications need a well-designed privacy-preserving framework such that users can benefit from ML without revealing their own models and training data. More recent approaches, including federated learning [55], have every user train their model locally and then exchange only model parameters with others, instead of the sensitive training data. Although this protects the local exchanges among users, it requires sophisticated approaches to security and privacy.

**Biometric Verification and Applications**—Biometric authentication helps in providing services such as access control to verify the individual’s identity based on their biometric traits [56]. These traits such as fingerprints, iris scans and behavioral characteristics are physically linked to an individual and unlike passwords or other identity documents, they cannot be easily forged or manipulated. Standard biometric authentication systems utilize a two-phase process consisting of registration, where users provide their template to the server, and a query phase, where a fresh template is provided for authentication. However, this process is *server-centric*, suffering from some inherent deficiencies such as users having to fully trust the server to properly handle their templates and the inherent noise of biometric template that may cause issues in the query phase of the process. This inherent noise may also obstruct the system from keeping the identity information in an encrypted form at all stages, providing an opportunity for adversaries to gain access to them. 

**Social and Mobile Networks**—While the current rolling out of 5G networks offers tremendous benefits to end users, information privacy has also raised serious concerns from users and service providers alike [57]. Balancing the trade-off between the strong confidentiality of user data and maintaining a low computation/communication overhead still remains a challenge, especially in big data applications. On the other hand, the amount of personal information stored on and shared through online social networks is immense, calling for an unprecedented level of security and confidentiality [58]. In the majority of these social networks, service providers make profits by generating advertisement revenue, where securing user privacy and generating accurate advertisements simultaneously can be tricky due to the involvement of user data decryption to extract keywords. 

Table 1 provides an overview of the representative literature considered in this work for different applications under the considered cryptographic primitives.

## 4. Attribute-Based Encryption

ABE is a public-key encryption scheme in which the user attributes determine their secret key and the resulting ciphertext. Thus, the decryption of a ciphertext is only possible if the set of attributes of the user key matches the attributes of the ciphertext. A review of the significant literature on ABE in terms of applications for the last decade is provided in Table 2. In the following, we review some works on the application of ABE in the IoT domain.

### 4.1. ABE for Constrained IoT Sensor Devices

**ABE Feasibility for IoT Devices**—The work of [59] explores the feasibility of ABE for some well-known IoT platforms and recommends its adoption for secure access control in IoT applications. A comprehensive analysis of the cost of ABE operations on resource-constrained devices is performed, where KP-ABE and CP-ABE are implemented on widely used IoT-enabling devices. The paper provides evidence of ABE feasibility for devices including Intel Galileo Gen 2, Intel Edison and Raspberry Pi Zero. The authors also present a smart healthcare use case to evaluate the proposed feasibility in real-world scenarios. The cryptographic operation performance is evaluated based on the assured security level (the number of bits used as primitives in cryptographic operations), where three security levels equivalent to the security provided by AES symmetric encryption using key lengths of 80, 112 and 128 bits are considered. The authors observe that the considered security level does not significantly impact memory usage, which is instead affected by the number of attributes in the policy. However, considering the execution time and energy consumption, the overall performance penalty is higher when the security level, instead of the number of attributes is increased. 

**ABE Feasibility for Constrained IoT Devices**—Further considering the adaptability of ABE for constrained IoT devices, [60] assessed the feasibility of ABE adoption in constrained devices characterized by limited capabilities in computing, storage and power. Three schemes corresponding to KP-ABE [23]. CP-ABE [18] and a recent KP-ABE for IoT [133] have been implemented and evaluated in terms of encryption/decryption time and energy consumption. The analysis presented by the authors highlights how ABE has a significant effect on the battery life of limited-power devices, which is significantly reduced when the number of employed attributes increases beyond 10. The authors specifically consider low-cost embedded devices with less than 1 MB of RAM and a micro-controller. The analysis shows that KP-ABE schemes are, in general, more efficient than CP-ABE ones, although CP-ABE schemes are considered more usable due to their association of access policy to data. It is also highlighted that instead of considering flat (in a flat policy, the decryption algorithm is always forced to visit all the leaves of the policy tree) policies, shaping the policies in many levels is good practice to improve the performance of CP-ABE schemes. 

**Lightweight ABE for IoT**—Addressing the energy needs of constrained IoT devices, the authors of [61] build their work on the premise that CP-ABE has not been designed taking into consideration the energy efficiency of most IoT devices. The authors propose to extend the basic CP-ABE scheme using effective pre-computation techniques. Although some previously proposed solutions, e.g., ref [134], aim to mitigate this concern by using online semi-trusted proxies to perform cryptographic operations on behalf of the data owner, the proposed scheme in this paper does not need the presence or management of any previously mentioned proxies. Rather, the work relies on the pre-computation techniques, avoiding any substantial changes to the security protocols. The proposed algorithm requires fewer computations, where in particular, there is no need for exponentiations or scalar point multiplications apart from small scalars. However, the memory consumption depends on the number of attributes, limiting the attributes in the access tree for a given device.

**Table 2:** Significant IoT-related literature for ABE in the last decade.

Ref.	Year	Author	Significance	Publisher
[135]	2022	M. Rasori et al.	ABE Survey for IoT	IEEE
[136]	2022	R. Imam et al.	ABE Review for Health Services	ScienceDirect
[137]	2021	Y. Zhang et al.	Combination of Blockchain and ABE for Access Control	MDPI
[138]	2019	S-Y Tan et al.	ABE Enhancement for IoT	IEEE
[139]	2018	P. Kumar P et al.	Survey on ABE in Cloud Computing	Elsevier
[64]	2017	Y. Rahulamathavan et al.	Blockchain Enhancement for IoT Based on ABE	IEEE
[59]	2016	M. Ambrosin et al.	ABE Feasibility for IoT	IEEE
[133]	2014	X. Yao et al.	Lightweight ABE for IoT	Elesevier
[65]	2015	M. Singh et al.	Secure MQTT for IoT	IEEE
[66]	2018	S. Belguith et al.	Cooperative Signcryption for IoT	IEEE
[68]	2019	M. Manna et al.	ABE for Industrial IoT	IEEE
[69]	2017	A. Alrawais et al.	ABE for Fog Security	IEEE

### 4.2. ABE for IoT Applications

**Secure User Access at IoT Middleware**—The authors in [62] proposed a CP-ABE scheme on the middleware layer in the IoT system architecture for user access control while reducing the complexity on the middleware. The authors aimed to provide data security along with access control on the middleware in IoT architecture, where the middleware only stores the encrypted data that are encrypted using the data owner’s access policy. The proposed system model contains four entities that are part of the access control scheme, the central attribute authority (CAA) which publishes public keys and a unique global identity (GID) to the users; data owners that define the access policy characterizing which subset of data each data user is allowed to access; data users who, with the right attributes, can access the desired information; and middleware, which stores encrypted data and acts as a data access provider to eligible users. Introducing the CAA between the middleware and data owner ensures the privacy of the user’s data. Apart from the inherent benefits of ABE, the scheme also provides resilience against collusion attacks. Since user’s GIDs are associated with their respective attributes, if two users have different GID approach CAA, it will identify that although they have the required set of attributes to fulfill an access policy, the attributes do not belong to the same GID. 

**Secure ML Engines in IoT Applications**—The work in [63] proposed a system to protect machine learning engines in an IoT environment without modifying the internal machine learning architecture. The premise builds on the security risks involved in the scenario where the computation process involves sensitive data in training and testing computations. The proposed scheme adopts the approach of a black box model where the machine learning engines are unchanged, i.e., the input and output of machine learning engines are preserved. To provide a secure access control, each computation request from the client to the server provides encrypted data with the appropriate access control attribute, otherwise the computation request is rejected. The authors proposed secure computation approaches for supervised learning models and predictions, where the scheme uses CP-ABE for key distribution and the key file is included in the device firmware. The provided results compare time and file sizes for both encryption and decryption processes, however, the computation is not performed for data with a size above 400 MB, since CP-ABE needs more allocated memory and processing power for higher data sizes. 

**Blockchain-Based IoT Ecosystem**—The work in [64] used ABE to address the privacy and confidentiality of the data shared in blockchain-based IoT ecosystems. It proposes a restructuring of the blockchain protocol to absorb ABE and provide an end-to-end privacy-preserving blockchain system. The use of blockchains in IoT offers major security advantages including the mitigation of data manipulation attacks, avoiding data tampering and trust building based on node reputation. The authors considered a decentralized ABE thus more than one AA issues credentials for miners and users, avoiding any single point of failure. Similarly to traditional blockchain applications, transactions are verified through AAs using ABE and new blocks are added after mining in a periodic fashion. To guarantee a stronger security, the blockchain protocol specifies the minimum number of miners for transaction verification and AAs will be forced to wait until the number of miners for an attribute surpasses the minimum requirement set by the protocol. 

**Secure MQTT for IoT**—Protocols such as Message Queue Telemetry Transport (MQTT) [140] are widely used in D2D communication in IoT for rapid developments, but lack security features. The authors in [65] proposed secure versions of these protocols where the existing security features are augmented with KP/CP-ABE, based on elliptic curve cryptography (ECC) [141]. The authors studied the suitability of ABE schemes for MQTT from an IoT perspective, and evaluate the performance of these secure protocols in IoT. A new published service ‘*Spublish*’ is described where the messages are encrypted using ABE, and suitability is provided based on lightweight ECC. The performance is evaluated in terms of the time taken to perform encryption, decryption, key generation and validation against a varying number of attributes with different key sizes. It is shown that a Secure-MQTT-based KP-ABE scheme is suitable for scenarios where the access policies are fixed and known a priori, and the requirement of an interactive public key generator (PKG) is feasible. On the other hand, secure-MQTT based on CP-ABE is more suitable for those deployments where devices can afford higher computing power and storage, and require dynamic access policies. 

**Cooperative Signcryption for IoT Applications**—The paper [66] presented a cooperative privacy preserving attribute-based signcryption scheme (C-ABSC) based on the constant-size attribute-based signcryption technique [142]. The main idea presented by the authors relies on the distribution of the signcrypting operation among different devices, with respect to the selected subsets of a general access predicate. Thus, each device signcrypts its input data and sends them to an untrusted aggregator (e.g., the edge node in a cloud scenario) who is capable of decrypting the received data only if a sufficient number of IoT devices cooperate. The proposed cooperative signcryption scheme does not reveal more information other than the authenticity of the information. The analysis of the proposed scheme further shows that the size of the signcrypted data does not depend on the number of attributes in the access policy. 

**Attribute-Based Encryption and Routing in ICN**—Due to data replication and dissemination, it is often difficult for the data owner to control data access in existing information-centric networking (ICN) implementations. The work of [67] enhances ICN’s ability to support data confidentiality by introducing ABE into ICN and making the approach specific to data attributes. The authors proposed an ABE and searchable data encryption (SDE)-based encryption scheme for content-centric data privacy in ICN, which offers fine-grained access control policies. The scheme facilitates large-scale applications by decoupling publishers and subscribers without any need to share keys, as is customary in symmetric encryption. Under the proposed approach, user privacy is preserved by encrypting subscription interests while routers can still forward encrypted data to subscribers. 

**ABE for Industrial IoT**—The authors in [68] proposed an ABE scheme suitable for industrial IoT applications, fABElous, aiming to minimize the encryption overhead in communication. It should be noted that the computation power for the execution of ABE is generally not a major concern, as it was shown to be suitable for IoT sensor devices [60], however, the overhead generated by ABE can be heavy for the communication protocol as it generates roughly 1 kB overhead per message. In the setting under consideration, a wireless sensor and actuator network (WSAN) is examined where sensors and actuators exchange encrypted information. Under the given threat model, an eavesdropper is unable to gain any information as they lack the symmetric key and the ABE decryption key. Similarly, if compromised, the architecture proposes that the sensors periodically refresh their symmetric keys, minimizing the information retrieved by an attacker. Although fABElous has a huge overhead as compared to no security, it has less communication overhead when compared to naive CP-ABE. Furthermore, as the number of data exchange executions increases, the overhead becomes lower and lower, and in its best-case scenario, fABElous has less than 50% of naive CP-ABE.

### 4.3. ABE for Fog and Cloud Applications

**ABE for Secure Fog Communications**—The authors of [69] proposed a key exchange protocol based on CP-ABE for establishing secure communications and enabling authentic and confidential communications among fog nodes. The proposed protocol establishes secure communications to exchange the shared key that is used to encrypt and decrypt the exchanged information. The fog nodes in the group can only obtain the shared key if they satisfy the policy defined over the set of attributes attached to the ciphertext. Based on the attribute set, a private key is issued for each fog node, while the cloud runs the encryption algorithm outputting an encrypted symmetric key which is broadcast to all fog nodes in the group. Upon receiving the encrypted key, each fog node runs the decryption algorithm using the assigned private key to extract the symmetric key. The proposed scheme is analyzed against collusion attacks, message authentication and forgeability, where the underlying structure based on CP-ABE provides the main defense. Apart from the proposed protocol, the authors also analyzed the performance and efficiency in terms of message size and communication overhead. The feasibility of the proposed scheme is demonstrated by implementing and comparing with an existing certificate-based protocol. 

**Secure Data Access in Fog Computing**—The article [70] proposed a secure and fine-grained data access control scheme with ciphertext update and computation outsourcing in fog computing for IoT. Since, for a secure ciphertext update, a user should be able to prove to the cloud service provider (CSP) that they possess valid attributes, the cryptographic technique of attribute-based signature (ABS) is used to help the CSP in user verification. Furthermore, most of the encryption, decryption and signing computations are outsourced from the end users to fog nodes. As a result, the computations for data owners to encrypt, and for end users to decrypt, re-encrypt and sign the data are irrelevant to the number of attributes in the policies. The security model is shown to be resistant and covers the aspects of data confidentiality, fine-grained access control, authentication and collusion. The performance efficiency of the proposed scheme is analyzed in terms of computational complexity for the data users, where it has been shown that the time for computations is small and constant, and the scheme can tolerate the increasing number of attributes well. 

**Secure Data Sharing in Cloud Computing**—The work of [71] addressed the challenges of simultaneous fine-grain access and data confidentiality in cloud-based data sharing by proposing a new attribute-based data sharing scheme. The proposed scheme was specifically designed for resource-limited mobile users in cloud computing. A majority of the computational tasks are eliminated by adding system public parameters and moving partial encryption computation to offline operations. Furthermore, a public ciphertext test phase is performed before the decryption phase, which eliminates most of the computation overhead due to illegitimate ciphertexts. The proposed scheme relies on an online/offline ABE scheme [143] that eliminates a majority of the computational load, allowing a resource-constrained mobile user to quickly transform a message into an ABE ciphertext. The proposed scheme allows anyone to check the validity of ciphertexts before performing an expensive full decryption.

### 4.4. ABE for Data Searching and Sharing

**Data User-Based ABE**—In the original ABE scheme, a central authority (CA) administers the system and generates secret keys for the users based on their attributes, where both the data owners (DOs) and data users (DUs) need to trust the CA. However, the CA is not always trustworthy and often, data users do not trust anyone but themselves. The authors in [72] proposed a new decentralized-ABE (DABE) scheme termed data-user-based ABE (DU-ABE), wherein DUs are not obligated to trust a CA. Instead, the DUs themselves take the responsibility and work as the authorities of an ABE system. In the proposed scheme, all the DUs possess attributes and run a DU-ABE system cooperatively to issue secret keys to themselves. In generating the authority setup, DUs are grouped according to their attributes and each member of the group generates a master key and a public key for that attribute. The DUs of different groups broadcast the public keys but save the master keys securely. Encryption setup by DOs takes into account the message, access matrix based on access policy, global setup and public keys of the relevant set of attributes, while for decryption, a DU employs the global setup and secret key related to a DU. For the generation of secret keys, all the DUs need to communicate with each other, which incurs a high communication overhead. 

**Secure ABE with Keyword Search**—The work of [73] presented a CP-ABE scheme with a fast keyword search which preserves the fine-grained access control inherited from ABE while supporting hidden policy and fast search. The proposed scheme supports AND-gate access policy with multiple attribute values, and uses multi-value-independent CP-ABE resulting in constant computation costs as compared to the previously proposed schemes. The authors proposed a multi-value-independent CP-ABE including authorized keyword search and a hidden policy. The search user obtains the secret key from the trusted authority, and given a keyword, the user generates a valid trapdoor and submits it to the cloud (only those users whose attribute set satisfies the specified access policy can generate a valid trapdoor). With the given trapdoor, the cloud server operates keyword search over the encrypted data and retrieves the corresponding information. 

**ABE with Personalized Search**—The authors in [74] addressed the design of an SE scheme termed AEPS which supports personalized search and fine-grained access control. A user interest model for individual users is proposed according to the department that the user belongs to and their search history, where in addition to offering the flexible search service in cloud computing, the scheme is able to return search results tailored to the user. Furthermore, AEPS achieves access control and search functionality at the same time, making it suitable for multi-user shared cloud search services. A personalized interest model is built to record and analyze the user’s department attributes and their search history. Using the access structure, the data manager controls the user’s access rights based on their attributes, while the search results are sorted according to the rank algorithm. The proposed scheme provides a solution of more flexible and personalized search for companies, hospitals and schools, while providing a solution to secure and inexpensive data storage in the cloud server. 

**Information Sharing in Tactical Mobile Networks**—The work in [75] presented a novel ABE cryptographic algorithm for battlefield tactical mobile networks (TMNs) with added capabilities for revocation, delegation and federation. The authors argued that it can serve as a foundation for a security infrastructure that allows efficient and effective information sharing on the TMN. The construction of the proposed algorithm is divided into a federated setup and key generation, key generation delegation as well as data distribution and access. The scheme is resistant against unauthorized access from a single user and against collusion attacks since ABE is inherently compatible with the TMN.

### 4.5. ABE for e-Health Applications

**Secure Medical Data Sharing using IoT Sensors**—The work in [76] proposed an efficient medical data sharing scheme that caters for smart terminals with limited computing power. Owing to user information leakage due to the attribute matching function, the use of attribute bloom filter (ABF) is proposed which hides the entire attributes in the anonymous access control structure. To generate the ciphertext more quickly, the use of online/offline encryption is proposed where the majority of the work needed in the encryption phase is completed before knowing the exact message. Furthermore, in the case of an increase in system users’ attributes, the proposed system does not need to be reinitialized, resulting in improved efficiency. Although hiding the values of attributes protects user privacy to a certain extent, even the names of attributes can leak some sensitive information. To cater for this, the authors propose to hide the attributes in an access control structure. For successful attribute assignment and gaining the secret key, both data owners and users should register and be authenticated with the attribute authority. Due to the use of pre-encryption technology, ciphertext is generated more quickly, which is well-suited for terminals with limited computing power. 

**User Study and Evaluation for ABE Adoption in Hospitals**—To study the integration of ABE in e-Health environments, the work in [77] conducted a study to identify use cases and requirements, and to learn the integration of ABE in IT processes. Based on the identified requirements through a focus group, an adaptive prototype implemented and evaluated through a cognitive walkthrough with usability experts. The target is to identify application scenarios and best-suited strategies to embed the useful features of ABE into the daily routine of a hospital. The authors proposed an architecture that is adaptive and matches the requirements of healthcare domain. The requirements derived from the focus group are taken into account for the development of a user management and container management (UMCM) endpoint, which deals with the authentication and authorization of users and also the validation of claimed email addresses that serve as part of user identity. The end users maintain a contact list and tags corresponding to identities with attributes, where the client application takes care of the policy authoring process.

### 4.6. ABE for Smart Cities

**ABE Utilization in Smart Cities Environment**—The use of cloud storage systems (CSS) generally lowers the capital and operating expenses, while guaranteeing high availability; however, there is also the risk of information disclosure to unauthorized entities. The work in [78] presented an encryption scheme for urban sensing which addresses the data storage concerns on the cloud while ensuring fine-grained access control by means of ABE. The work focused on a non-hierarchical ABE scheme with a single trusted third party (TTP), which generates decryption keys and provides them to users. The authors also proposed a key revocation method whose efficiency is based on a convenient attribute representation of the smart city, wherein IoT devices only execute the lightweight operations of symmetric cryptography. The proposed scheme also provides security against a variety of adversaries including the honest-but-curious CSS, an external adversary capable of eavesdropping traffic and compromising sensing devices, and a set of colluding users waning to illegally gain more authorizations.

## 5. Identity-Based Encryption

IBE is public-key encryption wherein a user generates a public key from a known unique identifier such as age or an email address or known location, and a TTP server calculates the corresponding private key from the public key. As a result, there is no need to distribute public keys before the exchange of encrypted information. A review of the significant literature on IBE in terms of applications for the last decade is provided in Table 3. In the following, we review some works on the application of IBE in the IoT domain.

### 5.1. IBE for IoT Applications

**Identity-based Authentication for IoT**—The work in [79] proposed an identification and authentication scheme for heterogeneity in terms of format, type and semantics for IoT networks based on a software-defined network (SDN) [144]. The central SDN controller translates the different technology-specific identities from the different silos into a shared identity based on virtual IPv6 addresses and authenticates devices and gateways through a hierarchical and distributed deployment. The key establishment method is based on elliptic curve cryptography (ECC) [141], while the root controller public key is assumed to be hardcoded in each thing when manufactured. The authors provided details of all the phases involved in the process of authentication, including public key certification, thing registration and the authentication phase. The evaluation of the proposed scheme shows that it is safe against masquerade, man-in-the-middle and replay attacks. 

**IBE for Post-Quantum Secure IoT**—The authors in [80] demonstrated that IBE has become practical for a range of embedded devices for the Internet of Things. The authors explored how different security levels and parameters for the underlying ring learning with errors (RLWE) assumption [145] will affect the implementation of IBE encryption and decryption in a range of typical IoT devices. Furthermore, the authors also proposed parameters for a pair of security levels that render an efficient IBE implementation via the number theoretic transform (NTT) [146] possible. In particular, the authors considered an ARM Cortex-M0 and an ARM Cortex-M4 for low-cost microcontrollers and a Xilinx Spartan-6 for FPGA implementation. The implementation results provided a detailed analysis of the applied techniques and algorithms, comparing the cycle counts and read-only memory (ROM) consumption, especially for the processes of encryption and decryption. The results suggested that IBE is practical for IoT devices as the performance is only slightly lower than the performance of RLWE Encrypt, with the added benefit of IBE’s simplified key management. 

**IBE for Anonymous Communication in IoT**—The article [81] proposed a scheme to ensure the privacy and anonymity of a communication system based on anonymous IBE, protecting the users’ metadata. In the proposed scheme, a user can send and receive the ciphertext in the same round of communication, which apart from causing sufficient confusion for the adversary, also results in improved efficiency. This work addresses the security goal of anonymous communication in three aspects, consisting of the message security, as well as the anonymity of both the sender and recipient. All the users follow a slotted time format, where the operations of encryption, upload, download and decryption are undertaken within pre-specified time slots. One downside of the proposed scheme stems from the fact that every user is required to send at least one message in each round of communication whether they want to have a communication or not.

**Table 3:** Significant IoT-related literature for IBE in the last decade.

Ref.	Year	Author	Significance	Publisher
[147]	2020	X. Jia et al.	IBE-Based Authentication for IoT	MDPI
[148]	2020	N. Farjana et al.	IBE for Security in Fog	Springer
[84]	2017	A. Karati et al.	IBE for Industrial IoT	IEEE
[149]	2017	J.Y. Kim et al.	Secure Management of IoT	ACM
[80]	2017	Tim Güneysu et al.	Lightweight IBE for Post-Quantum IoT Security	IEEE
[82]	2016	Y. Mao et al.	Fuzzy IBE for Secure IoT	Elsevier
[150]	2013	F. Li et al.	Integrating WSNs in IoT	IEEE
[141]	2012	B S Adiga et al.	IBE for M2M Communication	ACM
[79]	2016	O. Salman et al.	IBE-Based Authentication for IoT	IEEE
[81]	2018	L. Jiang et al.	IBE for Anonymous IoT Communication	Hindawi
[83]	2016	S. Sankaran	IBE-Based IoT Security Framework	IEEE

**Fuzzy IBE for Secure IoT**—The work in article [82] presented a fuzzy IBE scheme that is secure in the full model (i.e., the adversary can commit the target identity at any time) without random oracles, and at the same time, has a tight security reduction and short public parameters. Under the tight security reduction, the scheme does not need to enlarge the key and ciphertext size to obtain an increased security level. Due to these efficiencies of the proposed scheme, the authors believe that the scheme is more suitable for secure IoT communications. The authors also proved the semantic security of the proposed scheme under the modified bilinear Diffie–Hellman exponent (2-MBDHE) assumption, which is adapted from some of the previous [151] security constructions. 

**IoT Security Framework Based on IBE**—The paper [83] proposes a lightweight security framework for IoTs using identity-based cryptography (IBC). The authors developed a hierarchical security architecture for IoTs, and provided protocols for secure IoT communication such as for intra-domain and inter-domain communication, mutual authentication and revocation. In contrast to prevalent mechanism, the authors envisioned hierarchical topologies for IoT that can adapt to deployment at a massive scale. For the intra-domain communication, the authors adapt the Sakai, Ohgishi and Kasahara (SOK) scheme [152] for non-interactive key agreement. On the other hand, the mutual authentication scheme is a hybrid key management mechanism that uses IBE to set up pairwise symmetric keys between s and gateway nodes, and operates in bootstrapping, operational and post-operational phases. The scheme preserves data integrity by computing a message authentication code (MAC) on the pairwise symmetric key to provide an increased level of security, and it is shown that the scheme is scalable and incurs less overhead than traditional public key-based cryptography. 

**Identity Signcryption for Industrial IoT**—The authors in [84] presented an identity-based signcryption (IBSC) scheme using bilinear pairing for IIoT deployment. After studying two hard problems named modified bilinear Diffie–Hellman inversion (MBDHI) and modified bilinear strong Diffie–Hellman (MBSDH) under polynomial time intractable assumptions, the authors demonstrated through a rigorous security analysis that their scheme is provably secure based on the intractability of decisional-MBDHI and MBSDH assumptions. The scheme works on a multiplicative group elements, and proves its resistance in the formal security structure. The performance of the proposed scheme is illustrated considering the computational cost and the communication cost. Although the scheme is efficient, the cost during signcrypted IIoT data generation and verification can be reduced by eliminating the pairing overhead. Furthermore, the scheme does not support any revocation facility.

### 5.2. IBE for Smart Cities and Homes

**IBE for Smart Homes**—The work in [85] addressed the key challenges of the management flexibility and efficiency of computation and communication in providing a smart home system with confidentiality services. The authors proposed a lightweight encryption scheme for smart homes called lightweight encryption for smart home (LES), which is based on stateful IBE [153] combining IBE and stateful DH encryption scheme, encouraged to be used for resource-constrained devices. As an identity-based scheme, the public keys used for the scheme are merely identity strings, hence they do not need any certificates. The proposed scheme separates key encryption and data encryption in such a way that key encryption is performed less frequently. The authors provided a security definition for LES, and analyzed the security of the proposed scheme showing that it is IND-CPA secure in the random oracle model. The computational efficiency in the scheme comes from using the *state*, which is calculated only once between a smart home device and a user per communication. Numerical evaluation shows that in comparison to a stateless version of IBE, the encryption schemes accelerate nearly 10 times in the proposed scheme. 

**IBE for Smart City Information System**—In the work of [86], the authors propose a generic broadcast encryption scheme which is identity-based, and is from a generic anonymous IBE construction, providing confidentiality and anonymity simultaneously against chosen ciphertext attacks under DBDH assumption. The scheme also utilizes the strong one-time security notion for signature scheme (this captures the intuition that an adversary can request a signature σ on any single message *m* one time, after which the adversary should not be able to forge a pair of valid signature on another message; furthermore, the strong security requires that the adversary cannot generate a valid, but different, signature $\sigma^{\prime }$ on the same message *m*). The proposed generic identity-based broadcast encryption (IBBE) has a desirable property that its public parameter size and private key size are constant and that its decryption cost is independent of the number of receivers. Furthermore, the ciphertext is linear with the size of the receivers. Due to these characteristics, the proposed construction is deemed to be appropriate for smart city information systems. The security model of the proposed scheme builds on the IND-CCA (indistinguishability under chosen-ciphertext attacks), ANO-CCA (anonymity under chosen-ciphertext attacks) and WROB-CCA (weakly robust against chosen-ciphertext attacks) models. However, it should be noted that the construction is proven in the random oracle model.

### 5.3. IBE for Healthcare and Cloud Applications

**IBE-based encrypted traffic analytic for cloud computing**—The work of [87] used the concept of IBE with an equality test (IBEET) to propose a scheme for malware detection and the verifiability of encrypted data. The flow metadata, which includes the inbound and outbound bytes and packets along with the source and destination ports and flow duration in seconds, is used to compute a trapdoor for the detection of malware in encrypted data. The proposed technique builds on the work of [154] to allow the trapdoor function generation based on the flow metadata and byte distribution of encrypted data, where the metadata is sent to a remote MAP (malware analytic provider) server for verification. Based on the decision of the MAP server, which relies on the machine learning classifier-generated matches for a standard handshake scheme, the ciphertext is either forwarded to the cloud server for storage or rejected. Thus, the MAP server is presumed to possess the database records of standard handshake parameters and generated signatures between a client and the server. 

**Data Sharing in e-Healthcare Systems**—The paper [88] proposes a secure data sharing scheme for e-healthcare systems based on IBE with signatures to enable data sharing based on a user’s public identity. The scheme also ensures that data sharing is encrypted and authenticated such that only authorized uses are allowed to exchange any data. The data sharing is encrypted using AES, and authenticated using the BLS signature scheme (Boneh–Lynn–Shacham signature scheme that allows users to verify that a signature is authentic) [155]. The proposed protocol is divided into four algorithms, namely KeyGen, Extract, Encrypt & Sign, and Verify & Decrypt. The scheme only differs from original IBE scheme in the incorporation of AES, ensuring encrypted health information and verified recovered health data. The authors also showed the practicality of the proposed system through the computation times of encryption and signing, and decryption and verification on different platforms including the Arm Cortex Processor. 

**Data Sharing for Mobile Healthcare Social Networks**—The article [89] presented a secure data sharing and profile matching scheme for the mobile healthcare social networks (MHSNs) in cloud computing. The users are able to outsource their encrypted health records to cloud storage with an identity-based broadcast encryption technique, and share them with a group of doctors in a secure and efficient manner. The authors also present an attribute-based conditional data re-encryption construction which—should the pre-defined conditions in the ciphertext be satisfied—allows doctors to authorize the cloud platform to transform the ciphertext into a new ciphertext for any specialists that are authorized by the patient. The scheme also provides a profile matching mechanism in MHSN based on IBE with an equality test [154], where users can find friends and achieve authorization for the encrypted health records, with resistance against a keywords guessing attacks. The scheme is shown to be collusion-resistant against colluding doctors, while the scheme is also a one-way chosen-ciphertext-secure against a chosen identity attack.

### 5.4. IBE for Blockchain Privacy and Authentication

**IBE and Blockchain-Based Authentication**—The paper [90] proposes an improved key distribution scheme by integrating the techniques of blockchain [156] into the secure key issuing of IBE. The scheme implements mutual identity authentication between the communicating parties through integrating blockchain in the key issuing process. Users are treated as nodes of a blockchain, and are divided into the roles of supervision, production and protection nodes. The authentication process consists of key issuing and identity authentication to establish a secure communication channel. Furthermore, the role of the nodes are changed from time to time to effectively reduce the attack probability, where the change in roles is carried out through a consensus mechanism using the proof of vote (PoV). To prevent any network attacks, the scheme employs timestamps, random numbers and a hash algorithm in the process of identification. The analysis shows that the proposed scheme can effectively resist network replay attacks and DoS attacks, guaranteeing integrity and authenticity. 

**Data Privacy for Permissioned Blockchains**—The authors in [91] presented a practical scheme by adding IBE to blockchain systems, effectively improving the data privacy for non-transaction applications. The proposed approach has a high security level which can prevent both disguised and passive attacks, offering functionality, effectiveness and practicality in many applications for non-transactional scenarios. For many applications related to the real-world economy, such as quality tracking, copyright and supply chain finance, only data transfer is mainly needed which means that operations in consensus are primarily the consolidation of data. The authors first constructed a simple ID-based encryption privacy protection scheme that can be well applied to non-transaction scenarios in permissioned blockchains. In the proposed scheme, a user’s public key is generated through their identity, which simplifies the management and distribution of certificates in traditional PKI systems. Any new user joining the permissioned blockchain can obtain the encrypted key directly through their unique identity, which offers convenience in comparison to PKI systems.

### 5.5. IBE for Keyword Search and Biometric Verification

**Dual Trapdoor IBE with Keyword Search**—The work in [92] addresses an efficient search over encrypted data where previous schemes focus on supporting efficient and complex queries for the private key holder, while the authority cannot efficiently search the encrypted data. The authors introduced a new primitive named dual trapdoor identity-based encryption with keyword search (DTIBEKS), where the authority can use a peculiar means of producing an additional trapdoor allowing it to search for any identity’s encrypted data. The security of the proposed scheme is proven without random oracles, and is based on the methodology of dual system encryption [157]. The proposed scheme can be easily transformed into identity-based encryption with fuzzy keyword search by adopting a method similar to [158]. In the proposed primitive, two forms of trapdoors are considered, namely ‘identity-keyword trapdoor’ and ‘keyword trapdoor’, where the keyword trapdoor enables the cloud server to search for which data contain a given keyword regardless of who owns the data. 

**Biometric Identity Verification**—The article [93] described the first generic construction for multimodal biometric IBE considering two distance measures at the same time. In order to have a high recognition rate, and thus an increased possibility of decryption—even in the case of white noise—the proposed construction is based on two different biometric IBE systems encoding the same message. The scheme combines a fuzzy IBE-type scheme, which allows the use of biometric attributes as the identity instead of an arbitrary string, and the distance-based encryption (DBE) [159] with minimum overhead in terms of public parameters, ciphertext and private key size. Here, the authors described an IBE scheme denoted as ordFIBE, which is restricted for biometrics that can be represented as an ordered/grouped set of features. The proposed system can also be implemented as an ABE scheme, since the attributes of users can be grouped/ordered. If some of the attributes at the sender’s side do not match those on the receiver side, then due to the error-tolerance of the scheme, the receiver is still able to decrypt the ciphertext. The security of the scheme is analyzed under both the ROM and in standard model, and the efficiency is improved using an online/offline encryption scheme.

## 6. Searchable Encryption

SE allows a user to encrypt and send a message to an information receiver who can reassign it to a third party for searching the encrypted message for keywords without compromising the security of the encrypted message contents. A review of the significant literature on SE in terms of applications for the last decade is provided in Table 4. In the following, we review some works on the application of SE in the IoT domain.

### 6.1. SE for Fog and Cloud Applications

**Probably Secure SE for Cloud Storage**—The work in [94] addressed searchable encryption in cloud storage systems. The work first analyzed the security of a multi-user searchable encryption scheme presented by Wu et al. in [160] and it is shown that this scheme does not satisfy the invisibility of trapdoors. More precisely, it is shown that since the test algorithm can be executed by anyone, the challenger can guess the keyword trapdoor in at most two tests. Furthermore, the authors proposed a probably secure multi-user multi-keyword searchable encryption scheme, expanding the work presented in [161], which is a file-centric multi-keyword aggregate keyword searchable encryption scheme for IIoT, but suffers from the shortcoming of a data owner losing control over data due to the encryption execution at the same time. The presented scheme solves the problem of [161] by satisfying the indiscernibility of trapdoors against adaptive chosen keywords in the random oracle model. 

**Cross-Lingual Multi-Keyword Search over Encrypted Data**—The authors in [95] proposed a cross-lingual multi-keyword rank search (CLRSE) scheme which eliminates the language barriers and achieves semantic extension using the Open Multilingual Wordnet (OMW) [162]. The authors explored the problem of cross-lingual multi-keyword ranked search over encrypted cloud data, where the cross-lingual target query is built upon OMW. Through flexible keyword and language preference settings, as well as the automated calculation of preference scores for extended keywords for the semantic, the proposed scheme achieves intelligent and personalized sorting search and improves the accuracy of top-k search results. The proposed CLRSE scheme operates in different phases, including initialization, document outsourcing, cross-lingual query construction, trapdoor generation and top-k rank search. The security analysis of the scheme considers the confidentiality of the outsourced data, unlinkability of the query and privacy preservation as the key aspects. 

**Attribute-Based SE for Reliable Smart Grid**—The paper [96] introduced an attribute-based online/offline searchable encryption scheme where the encryption and trapdoor generation algorithms are separated into two different phases, and the message encryption and attribute control policy are performed in the offline phase. The authors combined the online/offline encryption scheme of [163] and the searchable scheme of [164] to propose this scheme. Based on the user’s attributes, the scheme provides secure data access to authorized users while protecting unauthorized users from accessing the data. The scheme thus achieves the properties of secure searchable ciphertext privacy, keyword privacy and a secure data access control. The proposed scheme offers the benefit of reducing the computation cost utilizing the online/offline phases. The proposed scheme is shown to be secure against both chosen plaintext and chosen keyword attacks. 

**Fog-Based Healthcare in IoT Networks**—The work in [97] proposed a fog-supported hybrid infrastructure where distributed fogs are deployed between IoT devices and cloud servers, providing temporary data storage, computation and analysis and network services. The authors designed a keyword searchable encryption scheme in the healthcare-related IoT–Fog–Cloud architecture, which ensures the security requirement that both data and keywords are protected from the cloud and the fog. The authors designed a fine-grained access control framework wherein a user should obtain their query capability authorization from a trusted authority and the fog through checking their attributes. To overcome the issue of resource-constrained IoT devices, the majority of the heavy computations of the proposed scheme are transferred to the fog and cloud, whereas only a small part is reserved for the users. The security analysis of the scheme demonstrates it to be secure under IND-CK-CCA attacks and to satisfy the trapdoor indistinguishability. 

**Dynamic SE with Privacy Protection**—The authors in [98] proposed a scheme to increase efficiency where the cloud is used to generate and store IoT-aggregated files. Forward privacy is achieved through a sublinear search efficiency by keeping an increasing counter for each keyword at an IoT gateway. The proposed scheme achieves forward privacy through the combination of locally stored state information and lightweight cryptography, whereas the cloud server is unaware of whether a newly added file consists of certain keywords except when the keyword is queried again. The proposed approach encrypts the combined increasing counter and the keyword together, making the server unable to link the keyword with the newly added file to any keywords in the cloud without knowing the secret key. In addition, a secure pseudo-random function is used to hide the connections with the generated tuples with the consecutive counter values.

**Table 4:** Significant literature for SE in the last decade.

Ref.	Year	Author	Significance	Publisher
[104]	2020	K. Zhang et al.	Lightweight SE for Industrial IoT	IEEE
[165]	2018	J. Ning et al.	Analysis of Passive Attacks on SE	IEEE
[166]	2017	G.S. Poh et al.	Detailed Review of SSE Schemes	ACM
[13]	2017	R. Zhang et al.	SE for Healthcare Clouds	IEEE
[102]	2018	L. Wu et al.	SE for Cloud-Based IoT	Elsevier
[101]	2017	M. Ma et al.	Certificateless SE for Industrial IoT	IEEE
[12]	2016	W. Yunling et al.	Survey on Main Techniques of SE	Springer
[167]	2014	C. Bösch et al.	Survey on Provably Secure SE Schemes	ACM
[168]	2013	E. Stefanov et al.	Dynamic SE Scheme for Small Leakage and Efficiency	Cryptology

### 6.2. SE for Secure Data Sharing

**Searchable Encryption for Data Sharing**—The paper [99] presented a scheme that enables authorized users to retrieve encrypted documents and verify the search results using a single aggregate key. The semi-honest-but-curious server is considered as a computationally bounded adversary, who may execute only a fraction of honest search operations. In the proposed verifiable searchable encryption with aggregate keys (VSEAK) scheme, the search keys and verification tokens are aggregated into one single key, avoiding the communication of massive key sets. The user is able to use the aggregate key to not only generate a single trapdoor as a keyword search query, but also to verify whether the server just conducts a part of computing for the search request. Thus, the data owner only needs to distribute a single aggregate key to other users to selectively share both search and verification privileges over their document sets. A majority of the user’s computations and storage are confidentially passed to the cloud server. Furthermore, an advanced scheme extending the work to multi-owner settings is also given which further reduces the users’ storage overheads. 

**Combining ABE and SE for Data Sharing**—The authors in [100] proposed a protocol based on the combination of SSE [169] and ABE [18] such that the main advantages of both are used. While the symmetric key needed for decryption is protected via a CP-ABE scheme, a user can directly search over the encrypted data through an SSE scheme. The considered threat model assumes that hardware integrity, physical security, network infrastructure and cryptographic security are all functioning in their standard way. Multiple users can efficiently and securely share files through the proposed protocol, and it can be considered as an independent contribution to the field of *hybrid encryption* since it combines both SSE and ABE schemes. In the proposed design, the authors separated the revocation functionality from the actual ABE scheme by proposing to use Intel’s Software Guard Extensions (SGX) to host a revocation authority in a trusted execution environment.

### 6.3. SE for IoT Applications

**SE for Industrial IoT**—The article [101] proposed the design of a secure channel-free certificateless searchable public key encryption with a multiple keywords scheme for IIoT deployment. The system model is composed of four standard entities including a cloud server, a data owner, a data receiver and a key generation center (KGC). The security of the proposed scheme is demonstrated in the random oracle model against two types of adversaries, where one adversary is given the power to choose a random public key instead of any user’s public key, while the second adversary is allowed to learn the system master key, where the security model is based on the indistinguishability of the chosen keyword attacks under the intractability of the standard BDH problem. The utility of this scheme is also demonstrated through its performance in terms of computational efficiency and communication cost. 

**SE for Cloud-Based IoT**—The work in [102] proposed a secure and efficient searchable encryption protocol using the trapdoor permutation function. The protocol is designed for cloud-based IoT, and compared to existing protocols, it incurs a lower computation cost at the expense of a higher storage cost. The protocol achieves inside keyword-guessing attacks (KGAs) resilience, forward privacy and file-injection attack resilience. This protocol uses neither bilinear pairing operation nor map-to-point hash operations, while the search time of the protocol is only dependent on the database update times. The system model is comprised of only three entities, namely the data sender (DS), data receiver (DR) and the cloud server (CS) where, apart from the traditional roles of DR and CS, DS is responsible for initializing the system, creating keyword indexes for files, and encrypting and uploading data. The computation and security parameters are compared to existing works based on bilinear pairing operation, the map-to-point, exponentiation, keyed hash function and scalar point multiplication. 

**Multi-user SE for Home IoT System**—The authors in [103] proposed a multi-user searchable encrypted voice scheme for voice systems based on home IoT systems, which enables users to send their voice commands to servers and retrieve each other’s voices. The proposed scheme employs long short-term memory (LSTM) networks algorithm to convert one user’s voice into another in order to improve the performance for multi-user retrieval. For enhanced security, the authors adopted an obfuscation function to hide the feature of the voice and DH algorithm to exchange parameters between users. The voice is divided into answers and queries where the answer part is encrypted and the query part is converted into features. The proposed scheme uses AES for encryption and decryption, where the small calculation overhead and suitability for large data blocks are the advantages while the key needs to be negotiated in advance and transmitted through a secure channel. The use of LSTM improves the precision and recall rate of the search while, to prevent the restoration of voice features, the obfuscation function helps process the mel frequency cepstral coefficients’ (MFCCs) features. 

**Lightweight SE for Industrial IoT**—The paper [104] proposed a lightweight searchable attribute-based encryption scheme that can significantly reduce the computing cost of IoT devices with the provision of multiple-keyword searching for data users. The proposed scheme is also extended to multi-authority scenarios to effectively generate and manage the public/secret keys in the distributed IoT environments. The proposed scheme is motivated by Green’s scheme [170] and Yang’s work [171], where the multi-keyword search in a distributed IoT environment was adopted. The proposed schemes offer the advantages of flexible access control, data confidentiality, lightweight decryption, accurate data retrieval and scalable key management. One of the scheme’s strengths lies in the preservation of the constant size of public parameters which do not vary with the number of attributes. Although the ciphertext of both the schemes is larger than most of the existing schemes, it does not affect the user experience since the ciphertext uploaded to the cloud does not require extra storage at IoT devices.

### 6.4. SE for Blockchain Privacy

**SE for Permissioned Blockchains**—The work in [105] built on the premise that existing approaches to search queries assume that the cloud server is “trusted-but-curious” or “honest-but-curious”, and to ensure greater levels of security, it should be considered malicious. The authors proposed an amalgamation of SE and permitted blockchains such that the client can place complete trust in the cloud server and the services it has to offer. The work presents a privacy-preserving framework that facilitates keyword search over the encrypted data stored on the blockchain network. Based on probabilistic trapdoors to resist distinguishability attacks and ensure high levels of security and privacy, the authors presented a privacy-preserving SE framework that facilitates the search over encrypted data stored on the permissioned and distributed ledger, i.e., hyperledger fabric [172], where the search over the blockchain is based on the SE scheme presented in [173]. Since the index table by default reveals the frequency of occurrence of an encrypted word within a document leading to a statistical analysis attack, the proposed algorithm masks these values to mitigate the risk of an attack and only reveals the presence/absence of an encrypted keyword in a document. 

**SE for Health Record Sharing**—The authors in [106] proposed a blockchain-based searchable encryption scheme for EHRs, where the index for EHRs is constructed through complex logic expressions and stored in the blockchain so that a data user can utilize these expressions to search the index. This gives the data owners full control over who has access to their EHRs, and the integrity and traceability of the data index is ensured through blockchain technology. In the proposed scheme, the real EHRs are stored on a public cloud server in encrypted form and users must be authenticated by the data owner to access these records. The scheme supports the complex query that allows healthcare agents to request permission to access and interact with the medical records, where the application uses a smart contract based on Ethereum [174]. The proposed scheme only focuses on the query accuracy, while it does not consider access to the data records. However, this can be achieved through existing file sharing schemes such as those based on ABE for fine-grained access control.

### 6.5. SE for Neural Networks and Geo-Referenced Data

**Encrypted Searchable Neural Networks**—The work in [107] proposed interpretable encrypted searchable neural networks to explore probabilistic query, balanced index tree construction and an automatic weight update in an encrypted cloud environment, resulting in an intelligent SE model. The probabilistic learning was used to obtain search ranking for searchable index, and a probabilistic query is performed based on the ciphertext index, which significantly reduces the computational complexity. More precisely, apart from the initial weight index generated by data owners, all the other automatic update operations are completed in an encrypted cloud environment, resulting in a lower computation, communication and storage overhead. The authors proposed a combination of adversarial learning [175] and automatic weight update in response to a user’s query of the latest dataset. Furthermore, to enable automatic weight update, a combination of backpropagation neural network [176] and Hopfield neural network [177] was proposed. Based on the neural network, the scheme sorts the network and employs probabilistic learning to obtain the query ranking for an encrypted searchable index. 

**SE for Geo-Referenced Data**—The authors in [108] presented different techniques to achieve range queries in SE, enhancing performance and reducing information leakage. The focus is on the case of one and two-dimensional range queries to an encrypted geo-referenced database in a client–server architecture. The authors provide techniques for SE to reduce the communication and computational costs of range queries based on over-covers, and to reduce information leakage under a symmetric key cryptographic scheme. The problem of searching encrypted data in the context of project CLARUS [178] is considered, which originally provides a framework for user-centered privacy and security in the cloud. For all the considered SSE schemes, the OXT scheme by Cash et al. [179] was chosen due to its suitability for static databases and highly efficient Boolean search causing moderate and quantifiable leakage to the server. In the considered data model, the client delegates an encrypted version of its dataset to the server, and afterwards, it retrieves a subset of the outsourced dataset through queries where query locations are considered to be rectangles.

## 7. Predicate Encryption

PE is a novel cryptographic primitive that provides accurate fine-grained access to encrypted data. In contrast to traditional public-key cryptographic systems, it has been widely adopted in point-to-point communication systems. A cryptographic system such as PE discerns the justification of information only being available to the users with access rights, providing more fine-grained control over the ciphertext. A review of the significant literature on PE in terms of applications over the last decade is provided in Table 5. In the following, we review some works on the application of PE in the IoT domain.

### 7.1. PE for Fog and Cloud Applications

**Privacy-Preserving Search in Cloud Storage**—The article [42] proposed a variant of symmetric predicate encryption which provides controllable privacy-preserving search functionalities including revocable delegated search and un-decryptable delegated search. These functionalities enable the owner of cloud storage to easily control the lifetimes and search privileges of cloud data. The proposed scheme is based on the work of [41], and introduces two new features including revocable delegated search and an un-decryptable delegated search. Here, the revocable delegated search makes it possible for the secret key owner to control the lifetime of the delegation, while due to the un-decryptable delegated search, a delegated person cannot decrypt the returned matched ciphertexts despite having the delegated search privilege. The considered system has three main roles, namely the secret key owner who wants to store sensitive data in the cloud and controls the encryption; a cloud storage service provider that stores data in the form of ciphertexts; and a delegated person who can obtain and decrypt the returned matched ciphertexts. The security of the proposed scheme is proven in detail through semantic security, attribute hiding and key confidentiality. 

**PE with Equality Testing in Cloud Computing**—The work in [109] presented the concept of attribute-hiding PE with an equality test by incorporating the notions of public-key encryption with the equality test and PE. Building on the idea, the authors presented an AH-PE-ET scheme that features constant pairing computations and minimal costs for decryption and testing. In the proposed scheme, a data receiver can calculate a trapdoor using their private key and deliver this trapdoor to an untrusted cloud server, who compares the ciphertexts from one receiver to the other receiver’s ciphertexts. The information about the provided trapdoor as well as the attributes associated with the ciphertexts are not disclosed to the cloud server during the comparison. The proposed scheme enables the cloud server to conduct an equivalence test on ciphertexts under various access policies. The scheme supports attribute-hiding as well as a more expressive access control. The authors also provide a rigorous comparison with existing ABE-ET schemes, in particular with [180,181], in terms of storage and communication cost, computation cost, functionality, security level and hardness assumption. 

**PE with Fine-Grained Search in Cloud Storage**—The authors in [110] proposed an efficient predicate encryption scheme and showed how to use it to implement a public key encryption with fine-grained searchable (PEFKS) capability mechanism. Furthermore, by combining PEFKS and PE into an integral encryption system, the authors are able to propose a privacy-preserving framework supporting an efficient predicate encryption with fine-grained searchable capability. The proposed scheme is computationally efficient since, for each attribute in the ciphertext and user’s key, there is only one group element and for each attribute in the decryption algorithm, only one pairing operation is required. The proposed PEFKS can not only search for the presence of multiple keywords in the ciphertext, but it can also evaluate the logical relations of these keywords. The efficiency of the proposed scheme is compared with the seminal works of [38,182], and shows that the scheme achieves a higher efficiency. 

**Enabled/Disabled PE in Clouds**—The article [111] proposed an enabled/disabled predicate encryption scheme that provides time-release services and data self-destruction. Given these characteristics, the receiver can set the readable and unreadable time of the files to be sent to the receiver, where the receiver can only read the file after the readable time. The structure of the file is destroyed after the unreadable time and the file then becomes unreadable. The time-release and data self-destruction properties are integrated with a predicate encryption that is based on the PE with the inner-product scheme of [183]. The proposed scheme can prevent the attacker from obtaining the ciphertext and performing crypt-analysis using any form of attacks on the ciphertext after the disabled time. Based on the proposed scheme, an extended scheme is also presented which, along with time-release services and data self-destruction, provides long message encryption and an undecryptable search. In the extended scheme, the length of the encrypted messages does not depend on the order of the group and the cloud server can only obtain the matched ciphertexts after the search.

**Table 5:** Significant IoT-related literature for PE in the last decade.

Ref.	Year	Author	Significance	Publisher
[184]	2021	Y.F. Tseng et al.	Efficient PE for IoT	IEEE
[109]	2018	J. Sun et al.	Attribute Hiding PE for Cloud Computing	IEEE
[116]	2018	S. Xu et al.	PE-Based Anomaly Detection in e-Health	IEEE
[114]	2017	C-I Fan et al.	PE for OSNs	IEEE
[110]	2016	X.A. Wang et al.	PE-Based Search for Cloud Storage	Elsevier
[111]	2016	S-Y Huang et al.	PE for Clouds	Elsevier
[113]	2016	W. Liu et al.	Public-Index PE for Mobile Access	Springer
[42]	2013	C-I Fan et al.	PE-Based Controlled Search in Cloud Storage	Elsevier

**Data Preprocessing for Fog Privacy**—The authors in [112] proposed a privacy-preserving data preprocessing scheme for fog computing in 5G network security. Specifically, the scheme is presented from the perspective of the quality of protection (QoP), aiming to preserve the security service option learned from attributes and to enable fog nodes to supply different levels of privacy protection services with different security demands from users. In the presented scheme, the end users have the options to contribute a list of attributes for their desired level of security protection. The proposed scheme intends to preserve the privacy of user data, by enabling fog nodes to supply different levels of privacy protection services with different security demands from users. The fog nodes are introduced to perform a privacy-preserving data preprocessing scheme for QoP. End users will conduct predicate encryption over these attributes, and fog nodes will provide the encrypted data with an additional and enhanced protection service based on the output run by the predicate utility function. The double-protected data will eventually be forwarded to and stored in a remote cloud.

### 7.2. PE for Social and Mobile Networks

**Online/Offline PE for Mobile Access Control**—The work of [113] proposed a general online/offline framework to address the expensive algebraic operations in public index predicate encryption (PIPE). The authors first proposed a generic transformation from a large universe PIPE which is secure against a chosen plaintext attack to online/offline PIPE under the same security model. To address the issue of generating a ciphertext without any knowledge of the associated ciphertext attributes in the offline phase, the authors presented a solution by identifying an attribute-malleability property in many LU-PIPE schemes. It is shown that with public-malleability, the security of the resulting OO-PIPE can be tightly reduced to the CPA security of the underlying LU-PIPE. Furthermore, the authors designed a generic transformation for CPA-secure LU-PIPE to OO-PIPE secure against an adaptive chosen ciphertext attack under the assumption that the underlying LU-PIPE has the specific properties of attribute-malleability and public-verifiability. To circumvent the issue of the online/offline mechanism implying forgery, the authors employed a universally collision-resistant chameleon hash [185]. The encryptor can replace the randomly encrypted ciphertext attribute in the offline phase with the target ciphertext attribute in the online phase, while an attacker cannot make such malleation. 

**Multireceiver PE for Social Networks**—The article [114] proposed a multireceiver predicate encryption (MRPE) scheme that is tailored for the online social networks (OSN) platform. In this scheme, the sender shares encrypted messages with a set of authorized receivers who can decrypt them, whereas the OSN provider can retrieve commercial keywords from the encrypted messages for advertisers, which improves the accuracy of advertisement without revealing the contents of the messages. The public parameters are defined by a third party, and the encryption process can be performed with a set of receivers. Since the public parameters are independent of the receivers, the length of ciphertexts can be compressed. Furthermore, the scheme allows each user to choose a part of their own secret value, while sharing the same public parameters. Here, the OSN provider is capable of finding corresponding keywords and producing customized advertisements.

### 7.3. PE for e-Health Networks and Authentication

**Application of PE to Anonymous Authentication**—The authors in [115] proposed an extension of predicate encryption named ‘delegate predicate encryption’ (DPE), where the user generates an encryption capability of a set of attributes, and then sends this capability to an encryption proxy. Using this capability, the proxy can encrypt an arbitrary message using these attributes without knowing anything about them. The proposed construction also satisfies the property named delegation transparency to the decryptor, which states that the receiver cannot tell whether a ciphertext is directly encrypted or via an encryption proxy. Thus, the anonymity of the provider and the authentication rules are well protected. The security requirement for DPE is defined by measuring the adversary’s advantage in winning a game, which is based on an extension of the game presented in [38] by adding the delegate encryption key query and a delegate encryption key corresponding to ciphertext in the challenge phase. 

**Anomaly Detection in e-Health Networks**—The paper [116] proposed a PE scheme for anomaly detection in networks applied to e-Health applications. Apart from the trusted authority, the system model consists of three distinct entities, namely a sender (provider); a receiver (examiner) which examines the critical component of a packet by validating the attribute vector with the predicate vector; and the malicious attacker, who forges the sensitive attributes of the created packets and deliberately falsifies sender’s packets with the contaminated elements. The contributions of the proposed scheme are measured in terms of confidentiality, privacy, efficiency and anomaly detection. The major contribution of this work comes from the use of the session key as a message in the encryption operation of PE system, which achieves information privacy and efficient cryptographic computation since, as compared to symmetric key encryption, here the session key is not pre-shared between the communicating parties. A case study in the form of evaluating an e-Health communication network is provided, where the performance of the proposed scheme is presented in terms of computational and communication overhead as well as anomaly detection.

## 8. Functional Encryption

FE is a public-key encryption scheme with different decryption keys allowing a user to learn specific functions of the encrypted data. The control that FE offers over which functions are allowed to be computed on the data by which user immensely benefits the data owner in multiple cases. A review of the significant literature on FE in terms of applications for the last decade is provided in Table 6. In the following, we review some works on the application of FE in the IoT domain.

### 8.1. FE for Data Sharing and Classification

**Proxy Re-Cryptography for Cloud Data Sharing**—Based on the work on proxy re-encryption called deterministic finite Automata-based functional proxy re-encryption (DFA-based FPRE) [186], the authors in [117] presented an outsourcing decryption scheme to increase the flexibility of users by delegating their decryption rights to a semi-trusted proxy. Based on the proposed construction, the decryption phase utilizes only one exponential operation instead of 12 pairing operations of the original work of [186], resulting in improved efficiency. This is due to the fact that the computation of bilinear pairings was considered a prohibitively expensive operation [187]. Only the user who has a DFA associated with their secret key can accept the key associated with the ciphertext and can efficiently access the encrypted data through the help of a semi-trusted proxy. 

**Data Ordering using FE**—The work in [118] proposed an improvised FE scheme for encrypted data ordering that takes as input multiple ciphertexts and orders them at the server site without compromising the data privacy. To achieve this, the authors formalized an order function using multi-input FE with obfuscation. Although data ordering can be achieved through order-preserving encryption (OPE) [188] and probabilistic predicate encryption [38], these approaches suffer from data privacy and efficiency issues and are not suitable for ordering operations at the server site. The proposed approach, MIFEO, runs the order function on multiple ciphertexts, and arranges them in an ordered set of ciphertexts. To use MIFOE in practical scenarios with existing cryptographic mechanisms, all incoming ciphertexts must be extended by associating an additional layer of encryption. 

**Private Data Classification using FE**—The authors in [119] used an instantiation of IPFE to perform the classification of encrypted data. The proposed method utilizes the fully secure FE for inner product functionality under the DDH assumption as given in [189]. Based on this, a multi-class prediction algorithm with encrypted input data is described. More specifically, the data item over which the prediction must be made is encrypted. From the encrypted data, integer inner products are extracted and are used afterwards to produce the class of the input data item. In the context of ML algorithms, the inner product can be viewed as a linear binary classifier. The learning process is kept secret and only the linear classifier’s coefficients are shared with the authority. Apart from the servers and users who have information to be kept secret but want to release classification results, a third party called the authority is introduced which handles the generation of FE keys and overlooks the process. The studied method ensures that the original image cannot be found from the inner product values.

### 8.2. FE for Machine Learning Applications

**Privacy-enhanced ML with FE**—The work in [120] introduced open source cryptographic libraries for FE. It presents, in detail, how FE can be used to build efficient privacy-preserving machine learning models, and provides an implementation of three prediction services that can be applied on the encrypted data. The paper addressed the lack of implementations of practical FE schemes that enable computation over encrypted data. Two full-fledged FE cryptographic libraries, named GoFE and CiFEr, are presented, which allow the library user to choose the underlying primitives when instantiating an FE scheme. Here, GoFE is implemented in GO, while CiFEr is implemented in C and aims at a lower level, possibly IoT-related applications. The three prediction services which are implemented using the developed libraries consist of an online privacy-friendly predictor of cardiovascular diseases, anonymous traffic heatmap service and image classification on encrypted data.

**Table 6:** Significant IoT-related literature for FE in the last decade.

Ref.	Year	Author	Significance	Publisher
[190]	2015	P. Ananth et al.	FE for Turing Machines	Springer
[191]	2015	K. Wrona	Military Perspective on Securing the IoT	IEEE
[123]	2018	O. Stan et al.	Tax Calculations Using FE	IEEE
[124]	2020	Y-B Son et al.	Energy Trading using Blockchain and FE	MDPI
[125]	2019	J-H Im et al.	Electricity Billing Using FE	MDPI
[126]	2020	H. Cui et al.	Outsourcing FE Using Blockchain	IEEE
[129]	2015	D. Sharma et al.	FE in IoT e-Health	Springer
[117]	2016	H. Abdalla et al.	FE for Public Cloud Data Sharing	IEEE
[118]	2018	D. Sharma et al.	Data Ordering with FE	IEEE
[119]	2017	D. Ligier et al.	Private Data Classification Using FE	Springer
[120]	2019	T. Marc et al.	Private ML Using FE	Springer
[121]	2019	R. Xu et al.	Privacy-Preserving Federated Learning using FE	ACM
[122]	2019	R. Xu et al.	Training NNs with Encrypted data	IEEE

**Privacy-Preserving Federated Learning**—In general, approaches that offer privacy guarantees incur a large number of communication rounds, substantially increasing the training time for FL systems. The authors in [121] proposed an approach for privacy-preserving FL employing a secure multi-party computation protocol based on FE, where a differential privacy mechanism is employed that defines a protocol from a multiparty FE scheme to mitigate the risk that curious aggregators and colluding participants will infer private information. The authors adopted the MIFE scheme of [192] with some modifications due to its computational efficiency. The approach also demonstrates a solution to the dynamic participant group issue, showing robustness to participant dropout or addition. To benchmark the performance, a convolutional neural network (CNN) was trained with the same topology as in [193] to classify the MNIST dataset of handwritten digits [194]. The evaluation results show that it can reduce the training time by 68% and the data transfer volume by 93% on average while providing a similar model performance and privacy guarantees as some existing approaches. 

**Training Neural Networks over Encrypted Data**—In the context of ML networks, it is beneficial to have a well-designed privacy preserving framework that does not require cloud and data owners to reveal their training models or information on the sensitive data. The work of [122] addressed this scenario where neural networks are trained over encrypted data without the overhead involved with interactive communication protocols, while supporting predictive analytics in a privacy-preserving way. The authors also constructed an FE scheme for basic arithmetic computations, termed FEBO, to support the requirements of the proposed approach. The intermediate data are not ciphertext in the hidden layers of the model, which intuitively indicates that the proposed approach can be expected to be more efficient than HE-based schemes. It should be noted that for training scenarios in ML, the HE-based approach does not allow the server to learn the prediction result, which the FE-based approach does.

### 8.3. FE for Smart Cities

**Tax Calculations in Smart Cities**—The work in [123] investigated how Inner-Product FE (IPFE) [189] can enable the design of a tax calculation system with built-in privacy. Based on FE, the authors proposed a new private-by-design taxation service for smart factories. They also proposed the application of this general approach for the use case of carbon tax where instantiations and experimental results under DDH and decision composite residuosity (DCR) assumptions are presented. The authors presented a carbon tax model for industries, taking into account the different fuel sources for the electricity they use, while keeping the amount of emissions that are produced private. The system architecture consists of the smart factory whose tax is calculated, a tax service entity in charge of the service proposing the taxation, and a qualified authority who not only is responsible for setting up the whole taxation strategy but also serves as the encryption key manager. The authors provided an IPFE construct based on [189] showing that for the subject computation of carbon tax, the inner-product primitive is sufficient since the assumed model is linear. 

**Energy Trading in Smart Grids using FE**—The authors in [124] proposed a peer-to-peer energy trading system on blockchains where the bids are encrypted and peer matching is performed on the encrypted bids by an FE-based smart contract. The system guarantees that the information encoded in the encrypted bids is protected but the peer matching transactions are performed by the nodes in a publicly verifiable manner through smart contracts. The proposed P2P energy trading system is based on a private Ethereum blockchain. The authors focused on achieving two goals, namely the transparency of the peer matching process and the confidentiality of the matching information. The system used the practical function hiding IPFE proposed in [195]. The prosumers remain anonymous to each other whereas the utility company knows their identity for accounting and billing purposes. Furthermore, the matching peers cannot repudiate their bids after the matching is complete. The feasibility of the proposed scheme is verified by implementing a prototype involving smart meters. 

**Electricity Billing Using FE**—Detailed power consumption data raise serious privacy concerns since personal data can be inferred from the energy usage profiles measured by smart metres. The article [125] proposes a privacy-preserving electricity billing method that does not sacrifice data quality for privacy, based on a novel use of FE. The proposed scheme is based on the scheme of [195], which develops on the function-hiding inner product encryption similar to the work in [196], and allows a smart metre to send the provider all of the measured data with full granularity and without privacy leaks. The smart metre encrypts the measured consumption and sends them to the electricity provider, who possesses a restricted decryption key, using which it can obtain the total consumption. The proposed system does not require any special purpose hardware and can be realized through a mere software update of the smart metre. Furthermore, it does not render the previous methods obsolete; rather, it can be combined for advanced services. 

**Decryption Outsourcing using Blockchains**—Since most FE schemes are built from bilinear pairings for which the computation is very expensive, a major issue in most FE schemes is efficiency. The paper [126] aims to design an FE with payable outsourced decryption (FEPOD). Leveraging the transactions involving cryptocurrencies supported by the blockchain technology, the payments in the FEPOD scheme are achieved through a blockchain-based cryptocurrency. This enables the data owner to pay a third party who correctly completes the outsourced decryption. However, there is always the issue of fairness between the user and the proxy, i.e., the user may refuse to pay even if they obtain a valid result. The proposed work is different from the works presented in [197] and [198] where [197] presents a fair exchange protocol to enable the exchange of a cryptocurrency payment for a receipt, whereas [198] addresses the need to integrate the FEOD scheme into the Bitcoin platform. The authors proposed a notion of FEPOD which allows anybody to check the correctness of the answer for the outsourcing computation task provided by an untrusted third party such that the payment can be processed through a blockchain-based cryptocurrency. 

**FE for UAV-Integrated Heterogeneous Networks**—The work in [127] proposed the use of FE in UAV-enabled HetNets to secure data against intrusion attacks. The process of implementing FE is proposed in two phases: first between UE and macro base station (MBS) and second between MBS and UE through UAVs. The work mainly focuses on the activation of an intrusion monitoring process and attacker ejection. Furthermore, a Bayesian game model was proposed to accurately detect the attacks with low overhead. Following the standard construction of FE, the work mainly proposes to secure the communication between the three parties, although no concrete construction is provided, and the security validation is proposed to be conducted through the AVISPA tool [199].

### 8.4. FE for Biometric Authentication, Healthcare and Cloud Applications

**Language Search over Encrypted Cloud Data**—The authors in [128] proposed the design of a novel privacy-preserving FE-based search mechanism over encrypted cloud data. The proposed new primitive supports an extreme expressive search mode, which is the regular language search. To facilitate this, the authors defined a new notion called searchable deterministic finite automata-based FE, which is a generalization of PEKS [39,200]. In the proposed construction, any system user can describe data to be shared with regular language in an encrypted form, where the language description can be arbitrary length. A valid data receiver can generate and deliver a search token represented as a DFA to a cloud server, such that the cloud server can locate the corresponding ciphertexts and return them to the data receiver. In the search phase, the server does not know anything about the search contents and the underlying data. 

**FE for e-Health IoT Systems**—To perform central processing, IoT systems require an automation tool or service at the central site which possesses collected data without compromising data security and privacy. The paper [129] presents a framework for the efficient utilization of centralized data while protecting data confidentiality and privacy in IoT infrastructure. The authors proposed a framework for the network layer of the IoT infrastructure to secure centralized data as well as utilize them for various analytical reasoning. Through the use of CP-ABE, privacy preservation with the access control of centralized data is proposed while with FE, various analytical functions can be executed on the encrypted data. A key feature of the proposed scheme is that since there is no dependency of any ciphertext on any function, the insertion of new ciphertexts as well as new functions is possible. On the other hand, a key limitation comes from the use of double encryption, where to create the FE ciphertext, already encrypted data (CP-ABE ciphertext) are used as the payload. 

**User-Centric Biometric Authentication**—The work in [130] proposed a user-centric biometric authentication scheme that enables end users to encrypt their own templates with a newly proposed light-weight encryption scheme. During the authentication process, all the templates remain encrypted and the server never sees them in their plain form. However, the server is able to determine whether the distance of two encrypted templates is within a pre-defined threshold. Under the scenario of both passive and active attacks, no critical information of the templates is revealed. The proposed scheme follows a compute-then-compare approach where the newly proposed primitive, threshold predicate encryption (TPE), can encrypt two vectors in such a way that the inner product of those vectors can be evaluated and compared to a pre-defined threshold. TPE uses similar techniques as in [201], however, the computational models as well as the security requirements are quite different. The proposed TPE can be used as a building block for various distance metrics involving encrypted data.

### 8.5. FE for Rights Managements and Searchable Encryption

**Secure Digital Rights Management**—The work in [131] proposed a novel Digital Rights Management (DRM) scheme based on a DFA-based FPRE scheme [186] which has been proven to be secure against CCA in the standard model. In the proposed scheme, a user with secret keys associated with their DFA accepts the ciphertext-associated string and can quite efficiently access the encrypted content with the help of the cloud service provider. The authors leveraged the DFA-based FPRE scheme to realize fine-grained access control over encrypted contents among a set of users, protecting the contents stored in a semi-trusted cloud environment and allowing flexibility in specifying the access rights of individual users. Through the use of computation outsourcing, the issue of high computation at the user side is also addressed. The work follows the scheme of [202] which allows the delegated key server to immediately revoke the attributes and malicious users. 

**Searchable Encryption using Multi-Input FE**—The authors in [132] proposed a secure and efficient searchable encryption scheme supporting multi-keyword search in a single-owner multi-user settings. The scheme is mainly applicable in cases where the number of keywords is limited but the number of files is large, such as sharing a comprehensive knowledge base in a certain field. In the proposed scheme, the cloud server is able to complete search processes with search tokens consisting of only two items, resulting in a significantly decreased communication and transportation overhead. The scheme achieves an efficient multi-keyword search through the use of an inverted index structure and super-incremental sequence. Furthermore, the scheme avoids per-query interaction between the data owner and data user, hence the data owner does not need to stay online for data users to search in their archives.

## 9. Open Challenges and Research Trends

Beyond the traditional secure computing methodologies, the developing domain of functional encryption has proven to be a promising avenue. Although crucial, contributions to cryptography design and implementation still need to be taken into account for further research projects. The main issues and interesting research paths for FE-based secure computing, in the majority of the IoT realm, are briefly discussed in this section.

**Inherent Factors affecting IoT Security**—security threats are a common issue that can seriously harm network infrastructure. IoT systems are vulnerable to security threats for a variety of reasons, including a the low processing power of smart devices, wireless connectivity, system openness and the physical accessibility of sensors, actuators and objects [203,204]. As a result, the three pieces of IoT hardware that may be exposed to frequent attacks are RFIDs, WSNs and clouds. Here, RFIDs are found to be the most susceptible technology since they are utilized in identifying and monitoring both people and items [205]. Furthermore, although IoT devices are manufactured with ease of use and connectivity in mind, they become vulnerable over time as attackers find new security issues to exploit. Regular patches and updates are needed to prevent these devices being exploited over time.

**Need for Secure Programming Protocol**—The majority of IoT devices are weak and susceptible to different types of malware due to their ease of accessibility and the limited computational resources at their disposal. Owing to a lack of transport encryption protocols, unsecured web interfaces, insufficient software security and authorization, this issue keeps on affecting and compromising the security of IoT nodes [206]. Thus, a device authentication and digital certificates approach can be adopted as a solution. More recently, the National Institute of Standards and Technology (NIST) released its recommendations [207] for device manufacturers to establish a security baseline. Furthermore, the traditional network reprogramming method has an authentication issue since it merely uses a data dissemination protocol to send codes in the network without running any authentication protocol [208], compromising network security. This requires the nodes to be aware of the validity of every code in order to avoid any malicious installation and to establish a secure programming protocol. A rogue node pretending to be a legitimate node in order to perform malicious activities poses another threat related to system and identity management.

**Threat Identification, Remediation and Multi-Key Attacks**—The development of lightweight cryptography for devices with limited resources is largely dictated by the key size and blocklength. The cipher text size increases with an increase in the key size, asking for more processing resources. One of the main problems concerning IoT security is a multi-key attack, which allows attackers to decrypt data using more than one key. In the event that the attackers obtain the key, the secrecy property may be jeopardized. The IoT system must identify and deploy countermeasures in order to address a security attack, and to continue functioning effectively [209]. In particular, after the system has been deployed, it must also be able to respond to new, unforeseen threats. These operations must be carried out lightly due to the constrained resources of IoT devices, and should utilize real-time calculations in order to contribute to a dependable and responsive system. For this objective to be successfully achieved, there is a need for procedures that teach developers to incorporate firewalls and intrusion prevention systems.

**Tighter Security Guarantees for FE**—Any secure computing protocols must address the crucial challenges of security and privacy assurances. The security of the underlying FE schemes determines how safe an FE-based secure computation can be performed. Existing FE methods with workable formulations often defend against the decisional Diffie–Hellman (DDH) assumption or selective indistinguishability under chosen-plaintext attack (IND-CPA). Even if such FE architectures meet security requirements in the majority of situations, they could not satisfy application circumstances, including those involving military applications, where strict security criteria/guarantees need to be met.

**Third-Party Infrastructures for FE Schemes**—Majority of FE schemes and related cryptographic primitives including ABE or PE rely on a trusted third-party authority (TPA) to supply a key service, with the exception of decentralized FE schemes. To enable the TPA-related FE-based secure computation protocols in the Internet environment, there are not a lot of generally accepted TPA infrastructures, apart from the commonly used certificate authority (CA) infrastructure [210]. The deployment of FE-based privacy-preserving applications in the actual Internet environment can be made easier, and the adoption of privacy-preserving applications can be hastened with the help of a transparent and generally trusted TPA infrastructure [211]. The implementation of such FE-based mechanisms will be more reliable with such an infrastructure for accountability and transparency. Although blockchains are perceived to eliminate the need for a TPA to enforce exclusion rights, and provide a system of universal access to knowledge and discoverability, recent works have started to look at the inclusion of TPAs. For example, the work of [212] proposed a framework that provides the transparency and trustworthiness of TPA and third-party facilities using blockchain techniques for emerging crypto-based privacy-preserving applications.

**Protection in Post-Quantum Era**—Recent advances in quantum computers are projected to impose severe threats to the security of widely used public-key cryptosystems and the communications that make use of it. It is therefore necessary to explore new post-quantum cryptographic algorithms and investigate architectures against strong attackers, especially those employing quantum computing [213]. Recent research works have investigated the application of potential post-quantum key encapsulation mechanisms and the digital signature algorithms identified in current NIST proposals [214]. There is always a risk of sensitive information being leaked as the function result in FE processing is communicated to the presumably sincere but inquisitive coordinator [121]. Therefore, it is necessary to investigate more robust security and privacy guarantees for safe computing protocols based on FE, including those for the post-quantum period.

In future works, we look to explore and utilize additional FE schemes, particularly multi-client and multi-input schemes that enable a broad range of applications such as querying encrypted databases, computing over encrypted data streams, and multi-client computation delegation. Furthermore, we intended to build and test function-hiding methods that will allow for privacy-preserving requests to prediction services.

## 10. Conclusions

In this survey, we looked at a variety of application areas where FE and various underlying cryptographic primitives have been used to enhance the security and integrity of user data including their identity and access rights, among others. We first presented a brief introduction to some of these primitives, including ABE, IBE, SE, PE and FE itself. After the overview of some of these potential applications, particularly those based on IoT sensors, we surveyed the recent literature utilizing these different cryptosystems in the aforementioned application areas. Although a lot of these schemes have been developed to address the broader areas of IoT and cloud computing, research in areas such as ML and biometric identification using state-of-the-art sensors is also being looked into by researchers. We aimed to provide the reader with a recent view of the developments in this growing area of research from the point of view of applications, such that it can inspire further interest. It should be noted that all the aforementioned primitives are provably secure and provide unquestionable security, which generally causes an efficiency barrier for various applications. For example, in a typical ABE implementation, the size of ciphertext is proportional to the number of attributes associated with the access policy, and the decryption time is proportional to the number of attributes used during decryption. Similarly, most current ABKS schemes incur large computation costs in the encryption and keyword search operations. In particular, when implementing an ABKS system inherited from ABE technology, the size of the ciphertext is proportional (at least linearly) to the number of attributes associated with the access policy. Future work should consider this important aspect in terms of trade-off between the security and efficiency for different cryptographic primitives, under various applications, and explore how the primitives might need to be modified to suit the application.

## Figures and Tables

**Table 1:** An overview of the representative literature discussed in this work.

ABE	Constrained IoT Sensor Devices [59,60,61]
	IoT Applications [62,63,64,65,66,67,68]
	Fog and Cloud Applications [69,70,71]
	Data Search and Sharing [72,73,74,75]
	e-Health and Smart Cities [76,77,78]
IBE	IoT Applications [79,80,81,82,83,84]
	Smart Cities and Homes [85,86]
	Healthcare and Cloud Applications [87,88,89]
	Blockchain Privacy and Authentication [90,91]
	Keyword Search and Biometric Verification [92,93]
SE	Fog and Cloud Applications [94,95,96,97,98]
	Secure Data Sharing [99,100]
	IoT Sensor-Based Applications [101,102,103,104]
	Blockchain Privacy [105,106]
	Neural Networks and Geo-Referenced Data [107,108]
PE	Fog and Cloud Application [42,109,110,111,112]
	Social and Mobile Networks [113,114]
	e-Health Networks and Authentication [115,116]
FE	Data Sharing and Classification [117,118,119]
	Machine Learning Applications [120,121,122]
	Smart Cities [123,124,125,126,127]
	Biometric Authentication, Healthcare and Cloud Applications [128,129,130]
	Rights Managements and Searchable Encryption [131,132]

## Data Availability

Not applicable.

## Abbreviations

List of abbreviations used in this work:

**Acronym **	**Description**	**Acronym**	**Description**
AA	Attribute Authority	KGC	Key Generation Center
AABE	Attribute-Based Encryption	KP-ABE	Key-Policy ABE
ABKS	Attribute-Based Encryption with Keyword Search	LSTM	Long Short-Term Memory
ABS	Attribute-Based Signature	MAC	Message Authentication Code
AES	Advanced Encryption Standard	MAP	Malware Analytic Provider
ASPE	Asymmetric PE	MANETs	Mobile Ad Hoc Networks
CA	Certificate Authority	MBS	Macro Base Station
CAA	Central Attribute Authority	CCA	Chosen Ciphertext Attack
MFCC	Mel Frequency Cepstral Coefficients	MHSN	Mobile Healthcare Social Network
CCA2	Adaptive CCA	MiTM	Man in The Middle
CNN	Convolutional Neural Network	ML	Machine Learning
CP-ABE	Ciphertext-Policy ABE	MQTT	Message Queue Telemetry Transport
CPA	Chosen Plaintext Attack	MRPE	Multireceiver PE
CSP	Cloud Service Provider	OPE	Order-Preserving Encryption
CSS	Cloud Storage Service	OSNs	Online Social Networks
D2D	Device to Device	PE	Predicate Encryption
DABE	Decentralized ABE	PIPE	Public Index PE
DBDH	Decisional Bilinear Diffie–Hellman	PEKS	Public Key Encryption with Keyword Search
DBE	Distance-Based Encryption	PKG	Public Key Generator
DO	Data Owner	NTT	Number Theoretic Transform
DU	Data User	RLWE	Ring Learning with Errors
DPE	Delegate PE	PKI	Public Key Infrastructure
DRM	Digital Rights Management	PoV	Proof of Vote
ECC	Elliptic Curve Cryptography	PRES	Proxy Re-Encryption with Keyword Search
EHRs	Electronic Health Records	QoP	Quality of Protection
FPGA	Field Programmable Gate Array	KGAs	Keyword Guessing Attacks
GID	Global Identity	QoS	Quality of Service
FE	Functional Encryption	SDE	Searchable Data Encryption
FHE	Fully Homomorphic Encryption	SDN	Software Defined Networking
FVM	Functional Virtual Machine	SE	Searchable Encryption
HABE	Hierarchical ABE	SSE	Symmetric SE
HE	Homomorphic Encryption	SPE	Symmetric PE
IBBE	Identity-Based Broadcast Encryption	SMC	Secure Multi-Party Computation
IBC	Identity-Based Cryptography	TMN	Tactical Mobile Network
IBE	Identity-Based Encryption	TPE	Threshold PE
IBSC	Identity-Based Signcryption	TTP	Trusted Third Party
ICN	Information-Centric Networking	UAV	Unmanned Aerial Vehicle
IoT	Internet of Things	IPFE	Inner Product FE
UMCM	User Management and Container Management	WSAN	Wireless Sensor and Actuator Network
SOK	Sakai, Ohgishi and Kasahara	ROM	Read-Only Memory
MQTT	Message Queue Telemetry Transport	MBDHE	Modified Bilinear Diffie- Hellman Exponent
